# Surrogate-guided self-play Monte Carlo tree search for antimicrobial peptide sequence optimization and *in vitro* antibacterial assessment

**DOI:** 10.1093/bib/bbag618

**Published:** 2026-11-13

**Authors:** Jang-Jih Lu, Chia-Ru Chung, Chen-Hao Chang, Li-Ching Wu, Justin Bo-Kai Hsu, Cheng-Yu Tsai, Tzong-Yi Lee, Jorng-Tzong Horng

**Affiliations:** Division of Clinical Pathology, Taipei Tzu Chi Hospital, Buddhist Tzu Chi Medical Foundation, No. 289, Jianguo Rd., Xindian District, New Taipei City 23142, Taiwan; Department of Computer Science and Information Engineering, National Central University, No. 300, Zhongda Rd., Zhongli District, Taoyuan City 320317, Taiwan; Department of Computer Science and Information Engineering, National Central University, No. 300, Zhongda Rd., Zhongli District, Taoyuan City 320317, Taiwan; Department of Computer Science and Information Engineering, National Central University, No. 300, Zhongda Rd., Zhongli District, Taoyuan City 320317, Taiwan; Department of Biomedical Sciences and Engineering, National Central University, No. 300, Zhongda Rd., Zhongli District, Taoyuan City 320317, Taiwan; Department of Computer Science and Engineering, Yuan Ze University, No. 135, Yuan-Tung Road, Zhongli District, Taoyuan City 320315, Taiwan; Division of Clinical Pathology, Taipei Tzu Chi Hospital, Buddhist Tzu Chi Medical Foundation, No. 289, Jianguo Rd., Xindian District, New Taipei City 23142, Taiwan; Institute of Bioinformatics and Systems Biology, National Yang Ming Chiao Tung University, No. 75, Boai Street, East District, Hsinchu City 300, Taiwan; Center for Intelligent Drug Systems and Smart Biodevices (IDS2B), National Yang Ming Chiao Tung University, No. 75, Boai Street, East District, Hsinchu City 300, Taiwan; Department of Computer Science and Information Engineering, National Central University, No. 300, Zhongda Rd., Zhongli District, Taoyuan City 320317, Taiwan

**Keywords:** reinforcement learning, multi-property optimization, deep learning surrogates, AlphaZero-inspired design, minimum inhibitory concentration

## Abstract

The rapid emergence of multidrug-resistant pathogens necessitates the development of novel therapeutic agents, yet the *de novo* design of antimicrobial peptides (AMPs) is challenged by the vast combinatorial search space and the difficulty of balancing efficacy with toxicity. To address these limitations, we introduce a reinforcement learning framework that employs surrogate-guided self-play Monte Carlo tree search to explore the peptide landscape. By integrating surrogate models for predicted antimicrobial activity, HemoDL-predicted hemolytic propensity, and ESMFold structural confidence, the system performs multi-property computational optimization and candidate prioritization. *In silico* analysis demonstrated that the optimized peptides adopt amphipathic α-helical structures with high predicted folding confidence and possess favorable docking scores against *Staphylococcus aureus* resistance targets, including PBP2a and MurA. To assess the biological relevance of the computationally prioritized candidates, 16 peptides were synthesized and evaluated by agar dilution against a 37-strain bacterial panel over a concentration range of 0.25–64 μg/ml. AMP2, AMP7, and AMP9 achieved complete inhibition of at least one tested strain, corresponding to 3 of 16 synthesized candidates (18.75%). AMP12 and AMP13 showed partial growth reduction without complete inhibition within the tested range, whereas the remaining 11 candidates showed no complete inhibition. These findings quantify the current gap between surrogate-defined computational enrichment and assay-specific antibacterial activity. The strain-dependent experimental outcomes further indicate that surrogate, docking, and structural scores do not fully capture all determinants of antibacterial efficacy. The experimental assessment was limited to bacterial agar-dilution activity; mammalian-cell cytotoxicity, wet-lab hemolysis, and peptide-stability assays were not performed. In conclusion, this study establishes a computational framework for the design of bioactive peptides and provides structural insights for bridging the gap between deep learning predictions and experimental outcomes.

## Introduction

Antibiotic resistance is an ancient and pervasive biological phenomenon that has been amplified into a modern clinical crisis by widespread antimicrobial exposure and rapid bacterial adaptation [1, 2]. The emergence of multidrug-resistant pathogens has underscored the necessity for therapeutic modalities that can augment—or, in certain instances, supplant—conventional small-molecule antibiotics. Antimicrobial peptides (AMPs) constitute an important class of innate immune effectors, exhibiting extensive antibacterial activity, numerous mechanisms of action, and a mechanistic basis that can mitigate the probability of expeditious resistance emergence when compared with numerous traditional antibiotics [3–5]. However, translating AMPs into deployable anti-infective agents remains challenging. This is due to the necessity of balancing antibacterial potency against hemolysis and other host-toxic liabilities, while also considering factors such as stability, manufacturability, and context-dependent activity, which further limit the development of these agents [4, 5].

Historically, the process of AMP discovery has evolved through two major phases. The initial phase involved the extraction and characterization of natural repertoires [6]. This was followed by a subsequent phase of optimization through combinatorial synthesis and high-throughput screening [7]. Concurrent efforts have led to the development of AMP mimics, which aim to enhance pharmacological properties and mitigate liabilities such as toxicity and instability [8]. The increasing number of approved peptide and protein therapeutics underscores the clinical feasibility of peptide modalities more broadly. However, it also highlights that successful translation typically demands extensive optimization and rigorous experimental characterization [9]. For AMPs, this challenge is intensified by the combinatorial size of peptide sequence space and the need to jointly satisfy multiple, often competing criteria such as activity, selectivity, and physicochemical robustness [4, 7].

To reduce the experimental burden of navigating these landscapes, protein and peptide engineering has increasingly adopted computational optimization strategies. Directed evolution provides a general framework for exploring sequence–function relationships through iterative mutation and selection. However, this process can be sample-intensive and may converge to local optima depending on library design and screening constraints [10]. Machine learning–assisted directed evolution advances this paradigm by training surrogate models to predict fitness and prioritize variants, thereby improving sample efficiency and enabling more structured exploration of sequence space [11, 12]. Meanwhile, reinforcement learning (RL) has emerged as a complementary approach for sequence optimization, particularly when the search process benefits from sequential decision-making and look-ahead planning. Self-play RL with Monte Carlo tree search (MCTS), a method popularized by AlphaZero for complex strategic domains [13] and subsequently adapted to protein engineering, offers an attractive mechanism to balance exploration and exploitation while optimizing toward a reward defined by predicted sequence properties [14].

In AMP engineering, deep generative and optimization frameworks have rapidly expanded the set of computational tools available for proposing candidate sequences. Generative adversarial network (GAN)-based approaches, including AMPGAN v2 [15] and Multi-CGAN [16], have been demonstrated to facilitate the learning of peptide-like sequence distributions and the incorporation of property conditioning. However, these approaches have been observed to potentially experience mode collapse and diminished diversity. Multi-objective strategies have been proposed to enhance the management of trade-offs between antibacterial activity and safety. For instance, HMAMP integrates hypervolume-driven objectives to collectively optimize multiple peptide properties [17]. Other pipelines, such as Diff-AMP, combine deep learning with RL components to support integrated generation, identification, prediction, and optimization [18]. Beyond GANs, Variational autoencoder (VAE)-based approaches—including HydrAMP and conditional denoising VAE frameworks—provide continuous latent representations that can facilitate interpolation and property-aware sampling [19, 20], while latent diffusion models have recently been applied to generate diverse AMP candidates, albeit often with higher computational cost [21]. Complementary tools such as PyAMPA provide high-throughput prediction and optimization components intended to streamline in silico screening prior to experimental testing [22]. Nevertheless, two critical limitations are observed throughout the current literature: Firstly, numerous methods are limited by distribution-restricted sampling or locally greedy optimization dynamics. Secondly, *in silico* objectives serve as imperfect proxies for experimentally measured antibacterial performance and selectivity. This motivates workflows that explicitly link computational optimization to standardized *in vitro* assessment.

The primary objective of this study is to develop a surrogate-guided self-play MCTS (SGSP-MCTS) framework for AMP sequence optimization. This framework is designed to navigate the AMP landscape under multi-property constraints and to prioritize candidates for experimental follow-up. The distinctive methodological contribution of SGSP-MCTS is the use of policy–value-guided MCTS to evaluate multi-step mutation paths before selecting each single-residue substitution. A fixed multi-property surrogate reward guides the search, while hard eligibility rules, classifier consensus, docking, and membrane molecular dynamics (MD) operate as subsequent candidate-prioritization stages. To incorporate structural considerations at the required for search-based optimization, rapid structure prediction is used to quantify folding confidence and to filter candidates for downstream characterization. Specifically, ESMFold is leveraged as a fast estimator of structural feasibility suitable for large-scale evaluation [23]. Finally, experimental susceptibility testing through minimum inhibitory concentration (MIC) assays is employed as the empirical endpoint for prioritized candidates, thereby enabling direct evaluation of how surrogate-guided optimization translates to measurable antibacterial activity.

## Materials and methods

Figure 1 provides overview of the integrated computational-to-experimental workflow. The schematic summarizes the principal data-source assignments, the SGSP-MCTS search-and-learning loop, the sequential post-generation candidate-triage stages, the structure-aware *in silico* prioritization process, and the completed agar-dilution antibacterial endpoint. Detailed inclusion criteria, mathematical definitions, reward coefficients, classifier benchmarking results, molecular-simulation settings, peptide-stock preparation, and agar-dilution procedures are provided in the corresponding Materials and Methods and Supplementary Methods sections.

**Figure 1:**
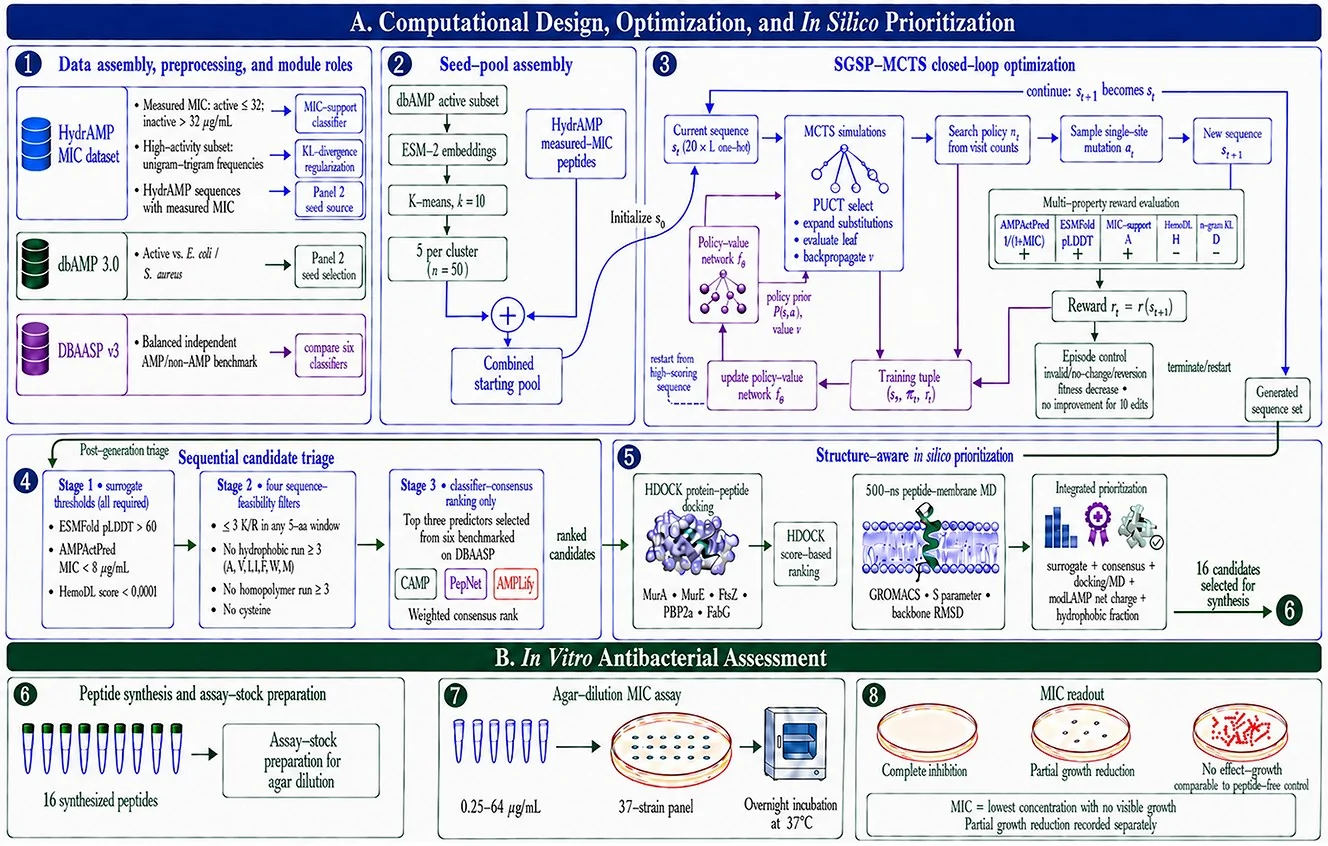
Overview of the SGSP-MCTS computational-to-experimental workflow for AMP optimization and in vitro antibacterial assessment. (i) HydrAMP, dbAMP 3.0, and DBAASP v3 provide module-specific resources for MIC-support classification, high-activity n-gram reference construction, seed-pool assembly, and independent classifier benchmarking. (ii) ESM-2 embeddings and K-means clustering yield 50 diversity-selected dbAMP representatives, which are combined with HydrAMP measured-MIC sequences to form the starting pool. (iii) Policy–value-guided MCTS initializes a seed sequence, constructs a visit-count mutation policy, samples single-site substitutions, evaluates the resulting sequence states using a fixed multi-property reward, and updates the policy–value network. (iv) Generated sequences undergo sequential post-generation triage consisting of surrogate-threshold screening, sequence-feasibility filtering, and post-filter classifier-consensus ranking. (v) HDOCK protein–peptide ranking, peptide–membrane MD, and physicochemical review prioritize 16 candidates for synthesis. (vi–viii) Synthesized candidates are prepared as assay stocks and evaluated by agar dilution against a 37-strain panel over 0.25–64 μg/ml. MIC is defined as the lowest concentration with no visible growth, while partial growth reduction is recorded separately.

### Data sources, inclusion criteria, and module-specific roles

Three peptide resources were used for distinct purposes in the workflow, as summarized in Fig. 1 (Panels 1–2). First, entries from the HydrAMP MIC dataset were included when an experimentally measured MIC was available [19]. These records were used to develop the auxiliary MIC-support classifier. Following the HydrAMP MIC classification setting, sequences with MIC ≤32 μg/ml were labeled active, whereas those with MIC >32 μg/ml were labeled inactive [19]. A high-activity subset of HydrAMP peptides was also used to calculate unigram, bigram, and trigram reference distributions. The KL divergence between these reference distributions and the corresponding distributions of generated sequences was incorporated as a regularization term during SGSP-MCTS optimization [24]. HydrAMP peptides with measured MIC values additionally contributed to the optimization starting pool.

Second, dbAMP 3.0 sequences annotated as active against *Escheri-chia coli* or *Staphylococcus aureus* were used for diversity-controlled seed selection [25]. Sequence-level representations were generated using a pretrained ESM-2 model [23] and partitioned by K-means clustering with k = 10. Five representative sequences were selected from each cluster, yielding 50 diversity-selected dbAMP sequences. These representatives were combined with the HydrAMP measured-MIC peptides to form the optimization starting pool. A two-dimensional t-SNE projection of the dbAMP embedding clusters is provided in Supplementary Fig. S1.

Starting-pool construction was based on measured MIC records, activity-related database annotations, and diversity-controlled sampling. Starting sequences were not required to satisfy all downstream surrogate-eligibility criteria before optimization. The ESMFold pLDDT, AMPActiPred-predicted MIC, and HemoDL thresholds were applied to generated sequences during the sequential candidate-triage stage described below.

Third, DBAASP v3 was used independently to benchmark the six AMP classifiers considered for post-filter consensus ranking [26]. A balanced AMP/non-AMP benchmark was used to compare ROC–AUC and summary classification metrics. CAMP, PepNet, and AMPlify were subsequently selected for weighted consensus ranking, as described in the Candidate Selection and *In Silico* Validation subsection and reported in Supplementary Fig. S2 and Supplementary Table S2. The DBAASP benchmark was used only for classifier evaluation and did not contribute to starting-pool construction or SGSP-MCTS reward calculation.

### SGSP-MCTS optimization framework

We formulated AMP sequence optimization as a single-player, sequential decision-making problem in which each state corresponds to a peptide sequence and each action applies a single-site amino-acid substitution. Because each trajectory proceeds through sequential single-site substitutions, the precise search path is conditioned on the selected starting sequence and its local sequence neighborhood. Optimization can be initiated from any sequence in the starting pool; no initial pLDDT or HemoDL requirement is imposed. At each visited state, the fixed reward components and MCTS search statistics determine the mutations explored from that sequence. This design directly addresses the “locally greedy” limitation of many prior optimization schemes by enabling explicit look-ahead planning: candidate mutations are not selected solely by immediate surrogate improvements, but by a tree search that balances near-term gains against longer-horizon opportunities through repeated simulations and value back-propagation, following the AlphaZero/EvoPlay paradigm adapted to protein engineering [13, 14].

Each sequence state ${s}_t$ was encoded as a one-hot matrix of shape *20× L*, where *L* denotes the number of mutable positions and 20 denotes standard amino acids. The action space was defined as the flattened *20× L* tensor, where selecting entry (*i, a*) corresponds to mutating position *i* to amino acid *a* (single-site mutation), consistent with prior self-play sequence optimization formulations [14]. When a mutation is applied, the one-hot column at the mutated position is updated to the chosen residue and all other residue indicators at that position are set to zero. Episodes proceed as a chain of mutations and terminate when (i) a mutation is invalid which means that no change or reversion to a previously generated sequence, (ii) the surrogate-derived fitness decreases relative to the previous step, or (iii) no improvement is observed for 10 consecutive mutation steps, at which point optimization restarts from a high-scoring sequence to continue exploration in a new neighborhood.

For each decision step *t*, we ran MCTS from the current root state ${s}_t$. During tree traversal, child actions were selected by maximizing a polynomial upper confidence trees (PUCT)-style score, as in AlphaZero [13] that combines exploitation via an action-value estimate with exploration via a prior derived from a learned policy:


$${a}^{\ast }=\arg \underset{a}{\max}\left(Q\left(s,a\right)+U\Big(s,a\Big)\right),$$

$$U\left(s,a\right)={C}_{\mathrm{puct}}\;P\left(s,a\right)\;\frac{\sqrt{\sum_bN\left(s,b\right)}}{1+N\left(s,a\right)}.$$

Here, $N\left(s,a\right)$ is the visit count for edge $\left(s,a\right)$, $Q\left(s,a\right)$ is the running mean value for that edge, $P\left(s,a\right)$ is the prior probability predicted by the policy head, and ${C}_{\mathrm{puct}}$ controls exploration. When an unexpanded (leaf) node ${s}_L$ was reached, it was expanded by adding candidate mutation children; the policy–value network ${f}_{\theta }$ evaluated the leaf to produce both an action prior and a scalar value estimate:


$$\left(p\left({s}_L\right),v\right)={f}_{\theta}\left({s}_L\right).$$

The predicted value *v* was then backpropagated along the visited path to update statistics:


$$N\left(s,a\right)\leftarrow N\left(s,a\right)+1,Q\left(s,a\right)\leftarrow Q\left(s,a\right)+\frac{v-Q\left(s,a\right)}{N\left(s,a\right)}.$$

This coupling of MCTS statistics with neural priors directly mitigates exploration failure modes observed in simpler RL-based AMP optimizers by preventing early commitment to locally attractive but globally suboptimal mutation paths and by maintaining principled exploration pressure in large action spaces [13, 14].

After completing MCTS simulations at root state ${s}_t$, we constructed a search-improved policy ${\pi}_t$ from visit counts and sampled the next mutation from:


$${\pi}_t(a)\propto N{\left({s}_t,a\right)}^{1/\tau },$$

where *τ* is a temperature parameter controlling exploration. The resulting tuple $\left({s}_t,{\pi}_t,{r}_t\right)$ was stored as training data, where ${r}_t$ is the surrogate-derived fitness of the post-mutation sequence which defined below. In this single-player “self-play” setting, the search distribution ${\pi}_t$ serves as a stronger supervisory signal than the raw network policy, enabling the model to iteratively bootstrap from its own search-improved decisions rather than relying on greedy mutation acceptance.

A central limitation emphasized in the Introduction is that *in silico* objectives can be weak proxies for experimental activity. To provide continuous guidance during MCTS exploration, we used the fixed multi-component surrogate reward described below. AMPActiPred and the MIC-support classifier supplied activity-oriented guidance, while ESMFold pLDDT and HemoDL-predicted hemolysis supplied auxiliary structural-confidence and hemolysis-risk guidance. Final candidate eligibility was determined separately through the strict post-generation thresholds described in the Candidate Selection and *In Silico* Validation subsection. Specifically, each generated sequence was evaluated by: (i) predicted antimicrobial potency via AMPActiPred (MIC-oriented prediction) [27], (ii) predicted local distance difference test (pLDDT) via ESMFold [23], and (iii) predicted hemolytic toxicity via HemoDL [28]. Because AMPActiPred first classifies whether a sequence is antimicrobial before returning an MIC estimate, MIC values can be undefined for sequences falling outside its AMP decision region. To prevent reward sparsity and search stagnation in those cases, we incorporated an auxiliary MIC-support classifier term *A* (probability of being a high-activity AMP) that continues to provide an activity-aligned signal even when MIC is unavailable, maintaining directional pressure toward antimicrobial regions of sequence space and addressing a practical failure mode of surrogate-only optimization loops. In addition, to reduce drift toward statistically implausible or non-functional motifs—another contributor to poor translation of purely computational designs—we penalized divergence from high-activity AMP motif statistics using an n-gram KL divergence term *D*.

All SGSP-MCTS optimization analyses reported in this study used the following fixed scalar reward:


$$\mathrm{R}=5\cdotp \frac{1}{1+\mathrm{MIC}}+0.02\cdotp \mathrm{pLDDT}\kern0.5em +\kern0.60em 1.0\cdotp \mathrm{A}-2\cdotp \mathrm{H}-0.1\cdotp \mathrm{D},$$

where MIC denotes the AMPActiPred MIC-oriented output, A denotes the probability generated by the auxiliary MIC-support classifier, H denotes the HemoDL-predicted hemolysis score, and D denotes the summed unigram, bigram, and trigram KL divergence from the high-activity AMP reference distribution. The coefficients were specified with consideration of the native numerical ranges and intended roles of the individual outputs. The reciprocal MIC term was assigned the largest positive coefficient to prioritize lower predicted MIC values. Because pLDDT is reported on an approximately 0–100 scale, its coefficient of 0.02 limits its nominal contribution to approximately 0–2. The auxiliary activity probability and HemoDL score are probability-like outputs on a 0–1 scale; coefficients of 1.0 and 2.0 preserve the activity-support signal and impose a comparatively stronger penalty on predicted hemolytic liability, respectively. The KL-divergence coefficient of 0.1 provides a mild regularization term that limits excessive departure from the motif distribution of high-activity AMPs. This fixed coefficient set was used for the reported optimization analyses. The fixed transformations, coefficients, output scales, and intended roles of the reward components are summarized in Supplementary Table S1.

The policy–value network ${f}_{\theta }$ was implemented as a lightweight 1D-CNN with a shared feature trunk and two task-specific heads, designed to maintain low-latency inference within repeated MCTS calls. The shared trunk consisted of three 1D convolutional layers (32/64/128 filters; kernel size 3; ReLU activations). The policy head comprised two additional 1D convolutions (80 then 20 filters; kernel size 3; ReLU) followed by a dense layer producing an *L× 20* softmax distribution over mutation actions. The value head comprised a dense layer (128 units; ReLU) followed by a single-unit tanh output producing $v\in \left[-1,1\right]$. Parameters were updated to align the value head with surrogate-derived rewards and the policy head with MCTS-derived search policies using:


$$\mathcal{L}\left(\theta \right)={\left(r-v\right)}^2-{\pi}^{\top}\log p+\gamma \parallel \theta{\parallel}^2,$$

where *p* is the predicted policy distribution, *v* is the predicted value, *r* is the surrogate fitness target, and *γ* is the ${\ell}_2$ regularization coefficient. This training scheme is standard in AlphaZero-style self-play learning and has been demonstrated to provide efficient sampling and improved search guidance in protein sequence optimization.

As summarized in Fig. 1, a seed sampled from the combined HydrAMP–dbAMP starting pool initializes the SGSP-MCTS optimization loop. Within this integrated workflow, MCTS and the policy–value network constitute the sequence-optimization engine: the policy–value network provides mutation priors and sequence-value estimates, whereas MCTS uses these outputs to conduct repeated look-ahead simulations over subsequent sequence states. AMPActiPred, ESMFold pLDDT, the MIC-support classifier, HemoDL, and n-gram KL divergence contribute to the fixed scalar reward evaluated during the search. The MCTS-derived policy and surrogate-derived reward are stored in training tuples for policy–value-network updating, and the resulting generated-sequence set is subsequently passed to the sequential post-generation triage described below.

### Candidate selection and *in silico* validation

Following sequence generation, candidates were processed through three sequential triage stages. Stage 1 required all surrogate eligibility thresholds to be satisfied: ESMFold pLDDT >60, AMPActiPred-predicted MIC <8 μg/ml, and HemoDL score < 0.0001. Stage 2 applied four sequence-feasibility filters: no more than three Lys/Arg residues within any five-amino-acid window, no hydrophobic run of three or more residues among A, V, L, I, F, W, and M, no homopolymer run of three or more residues, and no cysteine. Stage 3 applied a weighted classifier-consensus rank using CAMP, PepNet, and AMPlify, which were selected as the top three models from six classifiers benchmarked on DBAASP. The consensus score was used for post-filter ranking after the surrogate and sequence-feasibility criteria had been satisfied. Candidates were then subjected to a series of feasibility constraints in accordance with HydrAMP-style heuristic rules [19] . Sequences were excluded if they contained more than three Lys/Arg residues within any five-residue window, indicating an excessive local charge density. Additionally, sequences were excluded if they contained at least three consecutive hydrophobic residues, A, V, L, I, F, W, or M, or if they contained at least three identical residues, suggesting aggregation-prone runs. Furthermore, sequences were excluded if they contained cysteine residues, with the objective of avoiding uncontrolled disulfide chemistry. The sequence-feasibility rules and classifier consensus were applied after SGSP-MCTS sequence generation and did not contribute directly to the scalar reward or MCTS tree expansion. Subsequently, a classifier consensus score was computed by evaluating six AMP predictors: CAMP [29], AMPlify [30], PepNet [31], STM [32], AMPscanner [33], and AMPFinder [34]. The top three performers were then selected based on their performance on the independent DBAASP benchmark [26]. The final weighted ensemble utilized 0.356, 0.350, and 0.344 for CAMP, PepNet, and AMPlify, respectively, to generate a consensus AMP score employed for ranking and prioritization. Docking and membrane MD were subsequent prioritization procedures used to rank shortlisted candidates for synthesis.

For structure-based validation, the top-ranked candidates were evaluated by protein–peptide docking against five *S. aureus* resistance-associated targets, including MurA, MurE, FtsZ, PBP2a, and FabG. These targets were selected based on their roles in cell-wall synthesis and antibiotic resistance [35, 36]. The docking process was executed through the use of the HDOCK server [37], with the scoring and selection criteria being informed by prior AMP–target docking practices [20]. Receptor structures were obtained from the Protein Data Bank (PDB). These structures correspond to MurA: 3KR6; MurE: 4C13; FtsZ: 7OJZ; PBP2a: 1MWT; FabG: 3SJ7. Prior to analysis, the structures were preprocessed by water removal, residue refinement, and hydrogen addition in HDOCK [37]. Candidates exhibiting strong docking scores within the range of approximately ≤ −200 were prioritized for membrane-interaction assessment by MD. Peptide–membrane systems were constructed using CHARMM-GUI [38] with a POPE:POPG bilayer ratio of 120:60 lipids and 0.15 M KCl, using peptide starting structures predicted by AlphaFold3 [39]. Peptides were positioned at a distance of ~3.0 nanometers from the bilayer, with their helical axes oriented parallel to the membrane plane. Subsequently, a simulation was conducted for a duration of 500 ns at a temperature of 310 K under conditions of periodic boundary constraints. This simulation utilized the GROMACS [40] framework in conjunction with the CHARMM36/TIP3P configuration [38]. Membrane insertion was quantified by the S parameter, and structural stability was determined by backbone RMSD, in accordance with the analytical convention adopted in SELFPLAY [14]. SGSP-MCTS generated 19 268 candidate sequences. The recorded candidate-selection workflow progressively applied surrogate thresholds, sequence-feasibility rules, weighted classifier consensus, protein–peptide docking, membrane MD assessment, and physicochemical review, including net charge and hydrophobic residue fraction calculated using modlAMP [41]. This staged process yielded a final prioritized cohort of 16 peptides for synthesis and agar-dilution evaluation.

### Cross-method benchmarking design

Cross-method evaluation was organized according to the outputs available for each framework. Direct optimization behavior was evaluated for MCMC, PyAMPA, and SGSP-MCTS using the retained optimization records. All three methods were initialized from the same sequence, LFNRRGELRAIHKLKFWTLKDV, and were compared using optimization trajectories and the common surrogate-defined feasibility criteria of predicted MIC <8 μg/ml, predicted hemolysis score/probability <0.5, and both criteria jointly.

For sequence-level physicochemical comparison, isoelectric point, net charge, and hydrophobic residue fraction were evaluated for curated AMP and non-AMP reference sets and for available outputs from HydrAMP, Diff-AMP, latent diffusion, MCMC, PyAMPA, and SGSP-MCTS. For sequence-space and intra-method diversity analysis, ESM-2 embedding-based UMAP projections and pairwise Levenshtein distances were evaluated for HydrAMP, MCMC, PyAMPA, and SGSP-MCTS. The sequence sets in the diversity analysis were generated from the common high-predicted-hemolysis starting sequence FLPMLAGLAANFLPKIVCKITKKC.

### *In vitro* antibacterial assessment

Sixteen candidate peptides were synthesized. For agar-dilution plate preparation, each peptide was dissolved in sterile water at 8 mg/ml as a concentrated assay stock and divided into low-binding aliquots. Aliquots were stored at −80°C, and working aliquots were maintained at 4°C during plate preparation. The 8 mg/ml concentration enabled preparation of the final 0.25–64 μg/ml agar-dilution series. Long-term peptide stability in stock solution was not experimentally evaluated. Agar dilution MIC plates were prepared using Mueller–Hinton agar (MHA; 38 g/L), sterilized and cooled to 45°C–50°C prior to peptide addition. For each concentration, peptide solutions were mixed into 25 ml molten agar to achieve a two-fold dilution series spanning 0.25–64 μg/ml (details of stock-to-agar dilution calculations provided in Supplementary Methods—Peptide handling and dilution calculations for agar dilution MIC). Agar was poured into sterile plates, allowed to solidify, and used for inoculation.

A 37-strain panel was used, comprising 25 *S. aureus* isolates from prior collections, five reference strains, including *S. aureus* and Gram-negative controls, and seven clinical *Staphylococcus* isolates annotated as vancomycin susceptible/resistant (full strain list and identifiers in Supplementary Methods—Bacterial strain panel and inoculum preparation). All strains were cultured overnight at 37°C on blood agar plates, and single colonies were suspended in saline and adjusted to a 0.5 McFarland standard using spectrophotometry. MIC testing was performed using an agar dilution replicator workflow: inoculum suspensions were loaded into the sample plate, transferred to agar plates containing peptide concentrations using a 37-pin replicator (or dual 15-pin configuration), and plates were incubated inverted at 37°C overnight. MIC was defined as the lowest peptide concentration with no visible growth at the inoculation site. For candidate-level cohort reporting, an experimental hit was defined as a peptide that achieved complete inhibition of at least one tested strain within the 0.25–64 μg/ml concentration range. Partial growth reduction was recorded separately and was not used to assign an MIC or candidate-level hit status.

## Results

### Baseline surrogate profiles of the diverse starting pool

The initial pool used to seed SGSP-MCTS was first quantified using surrogate prediction. The construction of this pool was designed to achieve sequence diversity through the utilization of ESM-2 embeddings and clustering, as illustrated in Supplementary Fig. S1. Subsequently, the efficacy of the pool was evaluated using the same surrogate outputs that would later be employed for optimization purposes, including predicted MIC, predicted hemolysis probability, predicted AMP activity probability, and predicted folding confidence. The resulting distributions, as shown in Fig. 2, demonstrate that the predicted MIC values are concentrated in the low-to-moderate range, with most sequences falling below 20 μg/ml and a smaller number of higher-valued outliers, as illustrated in Fig. 2a. The predicted hemolytic probability is notably enriched near 0, yet it encompasses a distinct mass near 1, as shown in Fig. 2b, suggesting that the hemolytic liability remains heterogeneous even within an AMP-enriched starting set. Figure 2c demonstrates that the predicted AMP activity probability is notably skewed towards elevated values, while Fig. 2d shows the pLDDT ranges approximately from the mid-50s to the high-80s. These distributions show that the starting pool was enriched for activity-related properties but remained heterogeneous across the complete set of design criteria. Favorable predicted MIC and AMP-support values coexisted with variable HemoDL scores and ESMFold pLDDT values. Consequently, activity enrichment at initialization did not indicate that a seed simultaneously satisfied the activity, predicted hemolysis, structural-confidence, and sequence-feasibility requirements used for final prioritization. This residual multi-property heterogeneity provided the rationale for applying SGSP-MCTS to refine promising starting scaffolds through sequential mutation search.

**Figure 2:**
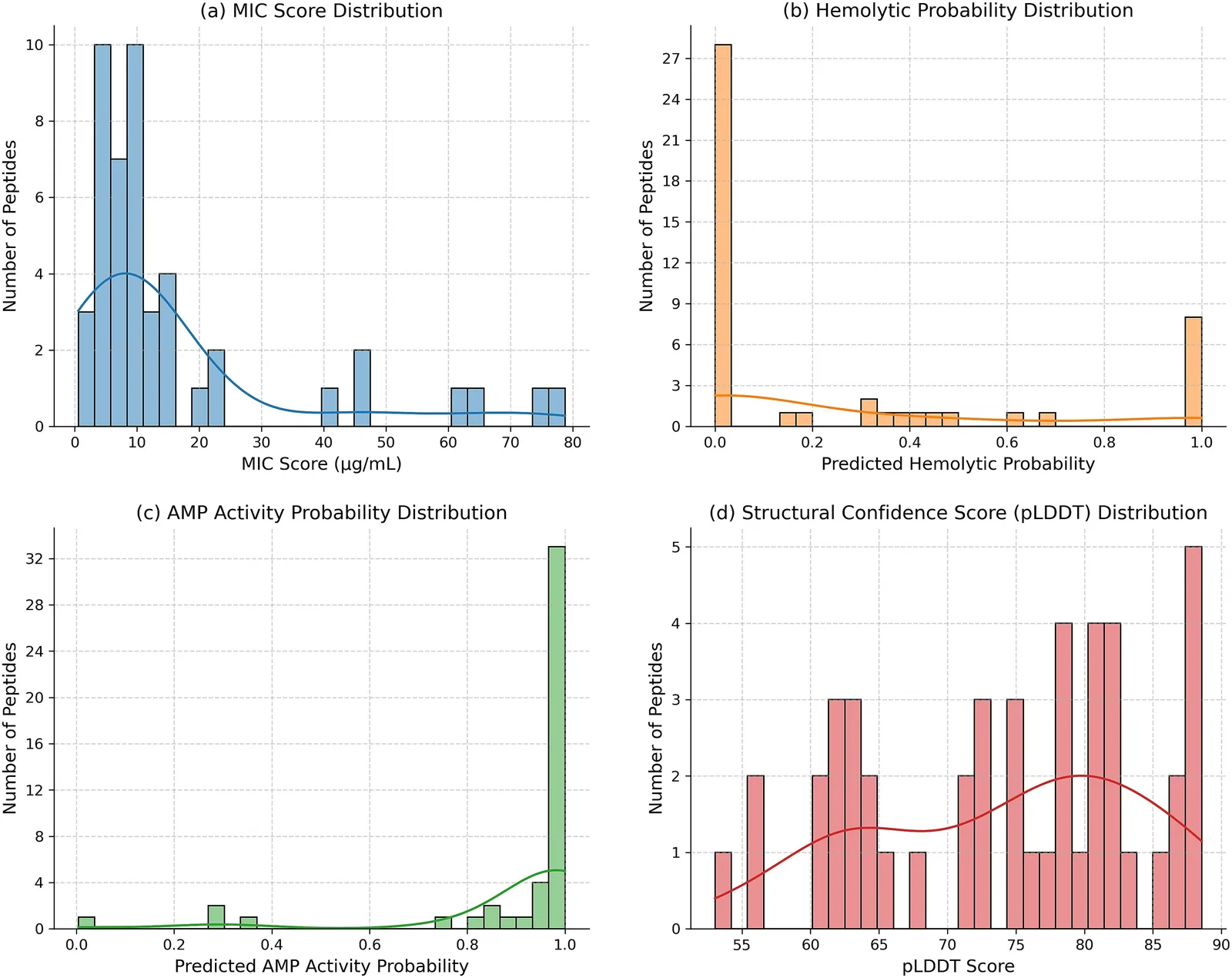
Surrogate-predicted property distributions of the activity-enriched and diversity-controlled starting pool. Histograms summarize (a) AMPActiPred-predicted MIC, (b) HemoDL-predicted hemolysis score, (c) MIC-support probability of high AMP activity, and (d) ESMFold pLDDT. The starting pool was constructed from HydrAMP MIC-dataset sequences and diversity-selected dbAMP representatives and was not required to satisfy all final post-generation eligibility thresholds. The distributions show favorable activity-related signals together with residual heterogeneity in predicted hemolysis and structural confidence.

### Representative SGSP-MCTS search trajectory from a high-predicted-hemolysis starting sequence

Figure 3 presents one archived 500-step trajectory initialized from FLPMLAGLAANFLPKIVCKITKKC, which was selected from the starting pool because of its high HemoDL-predicted hemolytic propensity. The starting sequence was not selected because all of its activity-related properties were poor; rather, it provided an example of a scaffold with a substantial predicted liability requiring multi-property refinement.

**Figure 3:**
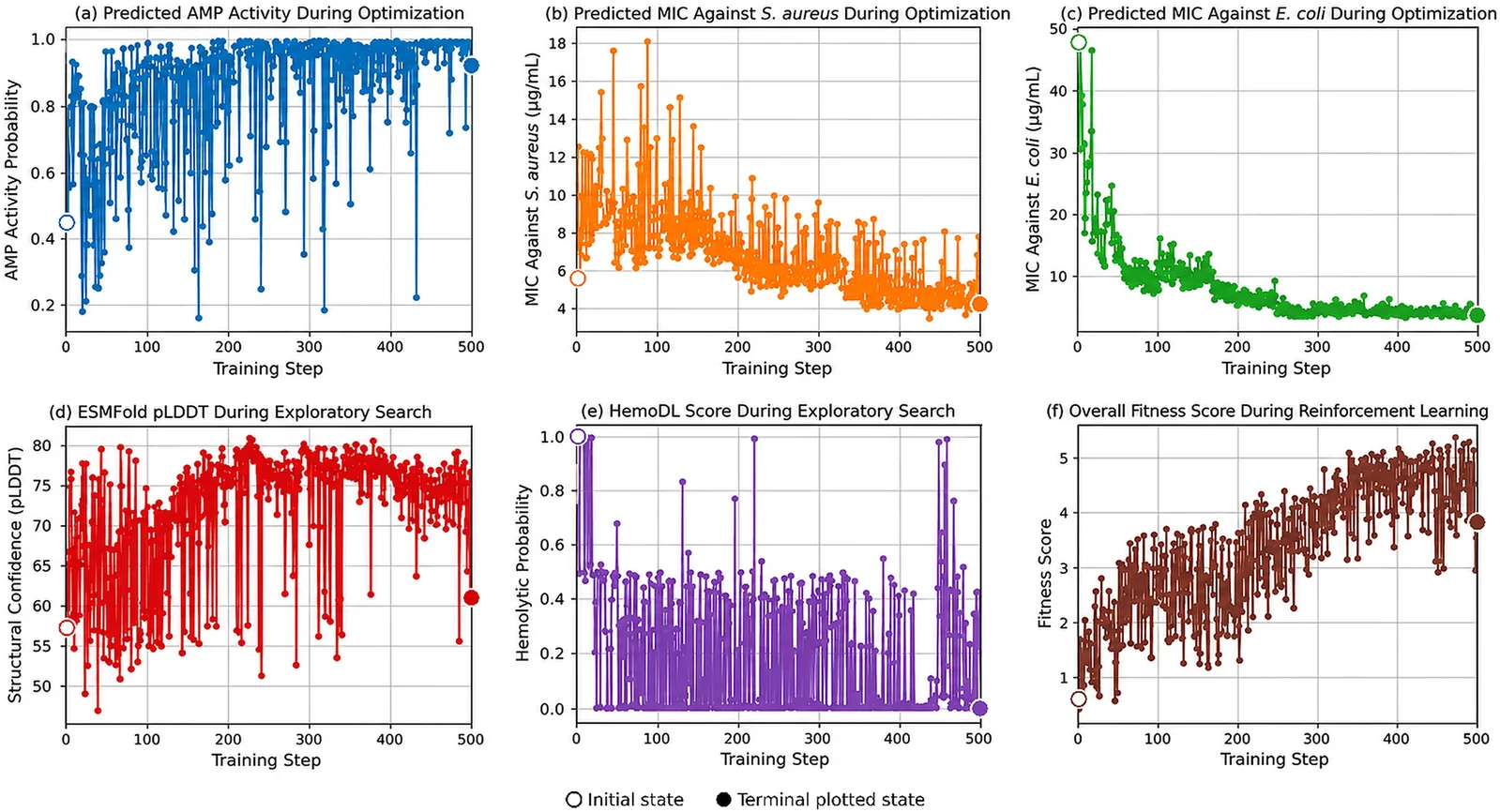
Representative exploratory SGSP-MCTS trajectory from a high-predicted-hemolysis starting sequence. The displayed 500-step trajectory was initialized from FLPMLAGLAANFLPKIVCKITKKC, a starting-pool sequence selected because of its high HemoDL-predicted hemolytic propensity. Panels show predicted AMP-support probability, AMPActiPred-predicted MIC against *Staphylococcus aureus* and *Escherichia coli*, ESMFold pLDDT, HemoDL score, and total scalar fitness. Enlarged markers identify the initial state at step 0 and the terminal plotted state at step 500 for direct within-trajectory comparison. The pLDDT and HemoDL traces represent intermediate exploratory states; final candidate eligibility was determined after sequence generation using pLDDT >60, predicted MIC <8 μg/ml, HemoDL score < 0.0001, and the sequence-feasibility rules. The terminal plotted state is not presented as a final synthesized candidate.

Large endpoint markers in each panel identify the initial state at step 0 and the terminal plotted state at step 500. Predicted MIC values decrease across the displayed trajectory, and the total scalar fitness generally increases. ESMFold pLDDT and HemoDL-predicted hemolysis fluctuate substantially among intermediate exploratory states. The endpoint markers enable direct within-trajectory comparison while preserving the complete unsmoothed time series.

This trajectory illustrates that the SGSP-MCTS search can continue to refine a seed even when some activity-related predictions are already favorable, because mutation selection is guided by the integrated reward and subsequent candidates must satisfy joint surrogate and sequence-feasibility criteria. The terminal plotted state represents the endpoint of this archived search trajectory and should not be interpreted as one of the final synthesized peptides. Final candidates were selected only after post-generation eligibility filtering, classifier-consensus ranking, docking, membrane MD, and physicochemical review. Figure 2 provides the complementary pool-level baseline, whereas Fig. 3 provides a single-seed within-trajectory comparison.

### Structural and physicochemical properties of optimized sequences

An evaluation was then conducted to analyze whether the optimized sequences retained structural plausibility and had similar AMP physicochemical properties. As illustrated in Fig. 4, representative AlphaFold3-predicted structures for optimized sequences demonstrate a predominance of α-helical conformations across the illustrated examples. The physicochemical comparisons are displayed in Fig. 5. Figure 5a and c demonstrate that curated AMPs have higher isoelectric points and more positive net charge in comparison to non-AMP controls. To provide a descriptive sequence-level comparison under common property calculations, we evaluated isoelectric point, net charge, and hydrophobic residue fraction for curated AMP and non-AMP references and for the available sequence sets from HydrAMP, Diff-AMP, latent diffusion, MCMC, PyAMPA, and SGSP-MCTS. Figure 5 shows that the SGSP-MCTS-generated sequences occupy cationic, AMP-relevant physicochemical ranges, including elevated isoelectric points, positive net charges, and moderate hydrophobic residue fractions. These distributions characterize the physicochemical properties of the evaluated sequence sets. Experimental antibacterial efficacy is reported separately in the agar-dilution assessment. Because the present study focuses on multi-property sequence refinement within an AMP-enriched design space, Fig. 6 compares the amino-acid composition of curated AMP sequences and SGSP-MCTS-generated peptides. This analysis evaluates compositional correspondence and residue-frequency shifts relative to established AMP sequence characteristics. The SGSP-MCTS cohort shows a higher lysine frequency, a lower arginine frequency, and differences in glycine, proline, and several additional residues relative to the curated AMP cohort. These observations characterize the composition of the optimized sequence set within the AMP sequence space. AMP-versus-non-AMP discrimination is evaluated independently using the DBAASP classifier benchmark in Supplementary Fig. S2 and Supplementary Table S2, while Fig. 5 provides complementary non-AMP reference distributions for isoelectric point, net charge, and hydrophobic residue fraction.

**Figure 4:**
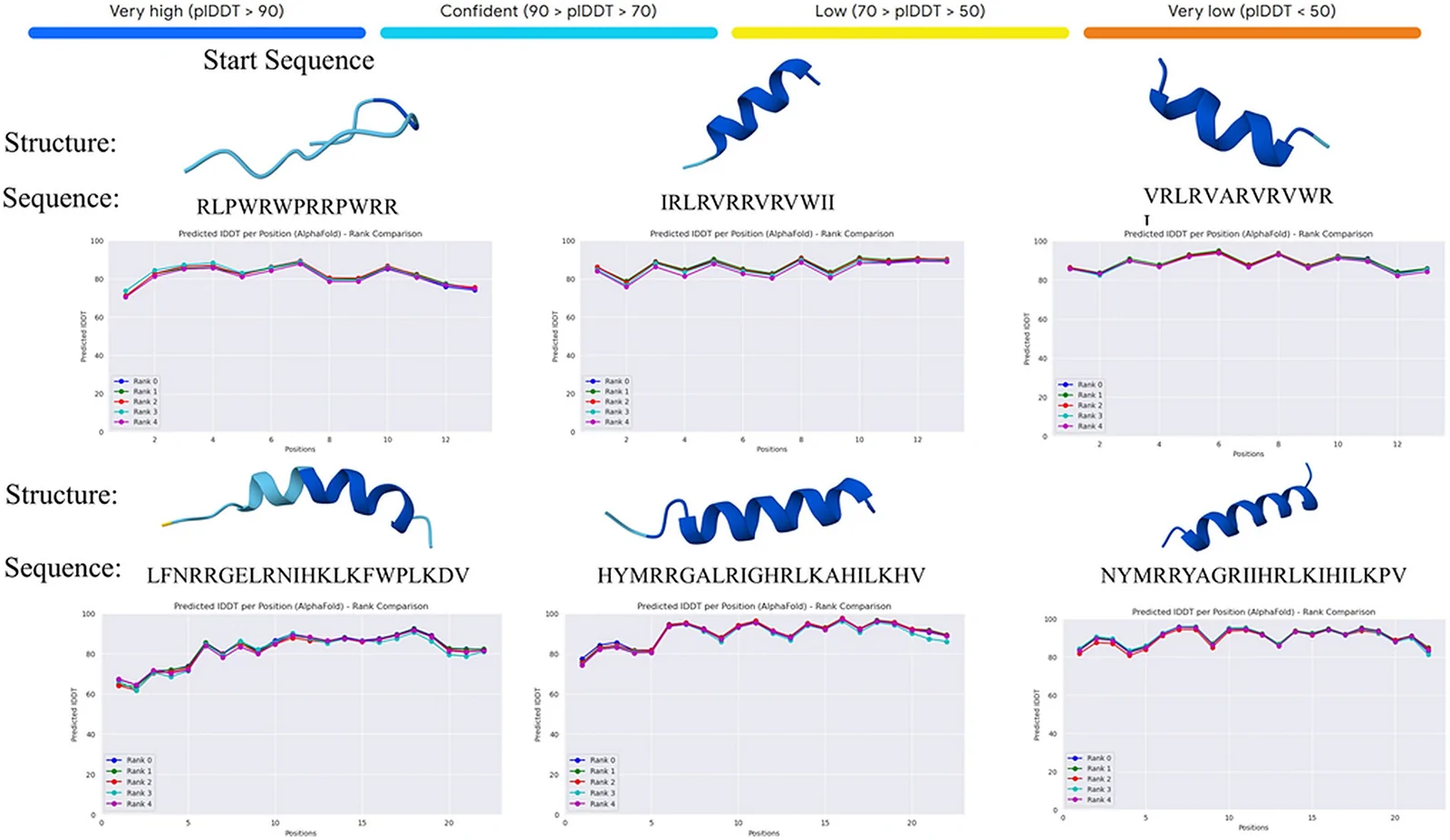
Representative structural models for peptides sampled during optimization. Cartoon representations show AlphaFold 3–predicted peptide structures for representative sequences sampled during optimization. Per-residue confidence is reported as pLDDT, and the corresponding confidence profiles across ranked models are shown beneath each structure.

**Figure 5:**
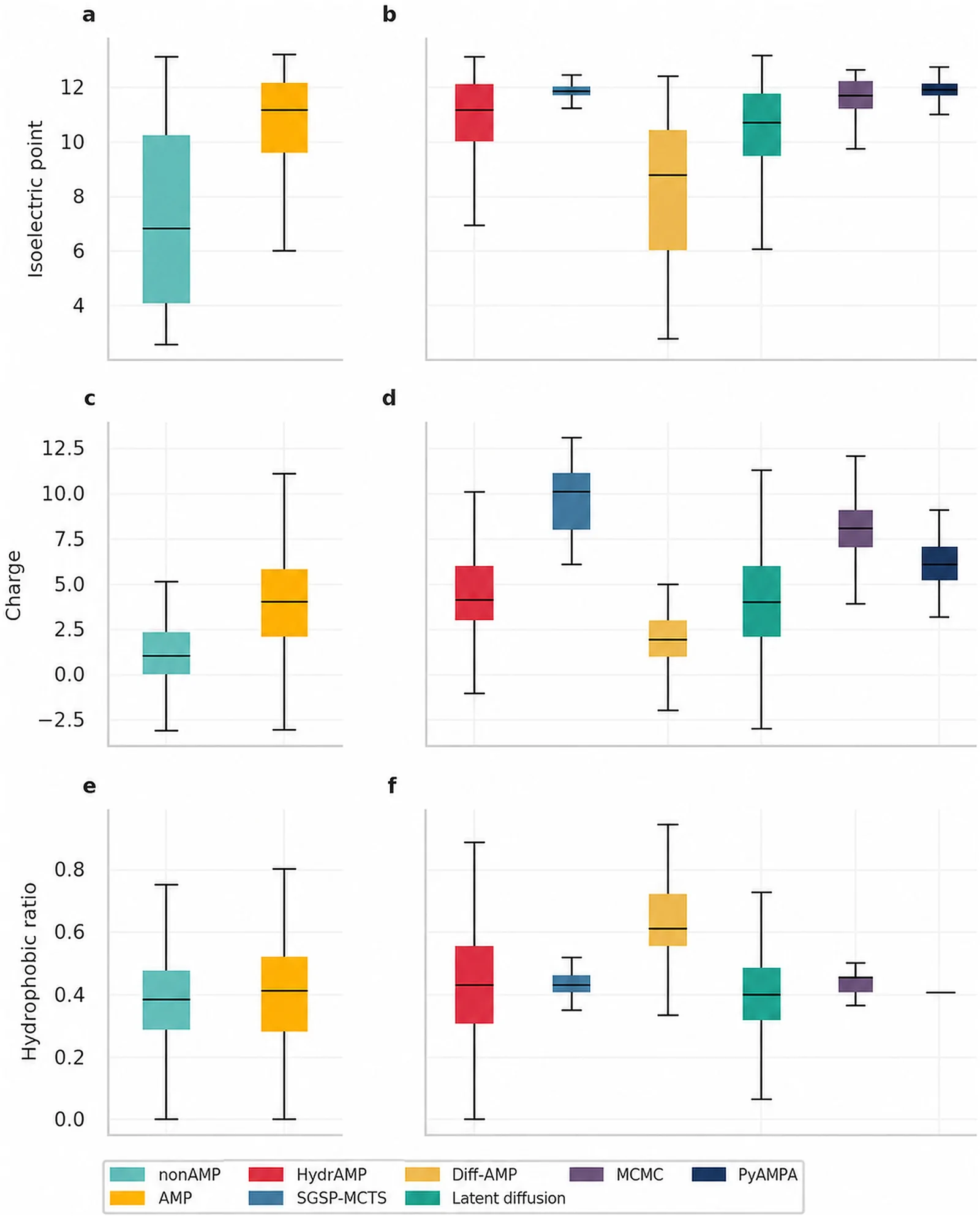
Descriptive physicochemical comparison of generated peptide sequence sets and reference cohorts. Boxplots show isoelectric point, net charge, and hydrophobic residue fraction for curated AMP and non-AMP reference sets and for the available sequences from HydrAMP, Diff-AMP, latent diffusion, MCMC, PyAMPA, and SGSP-MCTS. All displayed properties were calculated using common computational definitions. These distributions characterize sequence-level physicochemical properties and do not represent experimental antibacterial hit rates.

**Figure 6:**
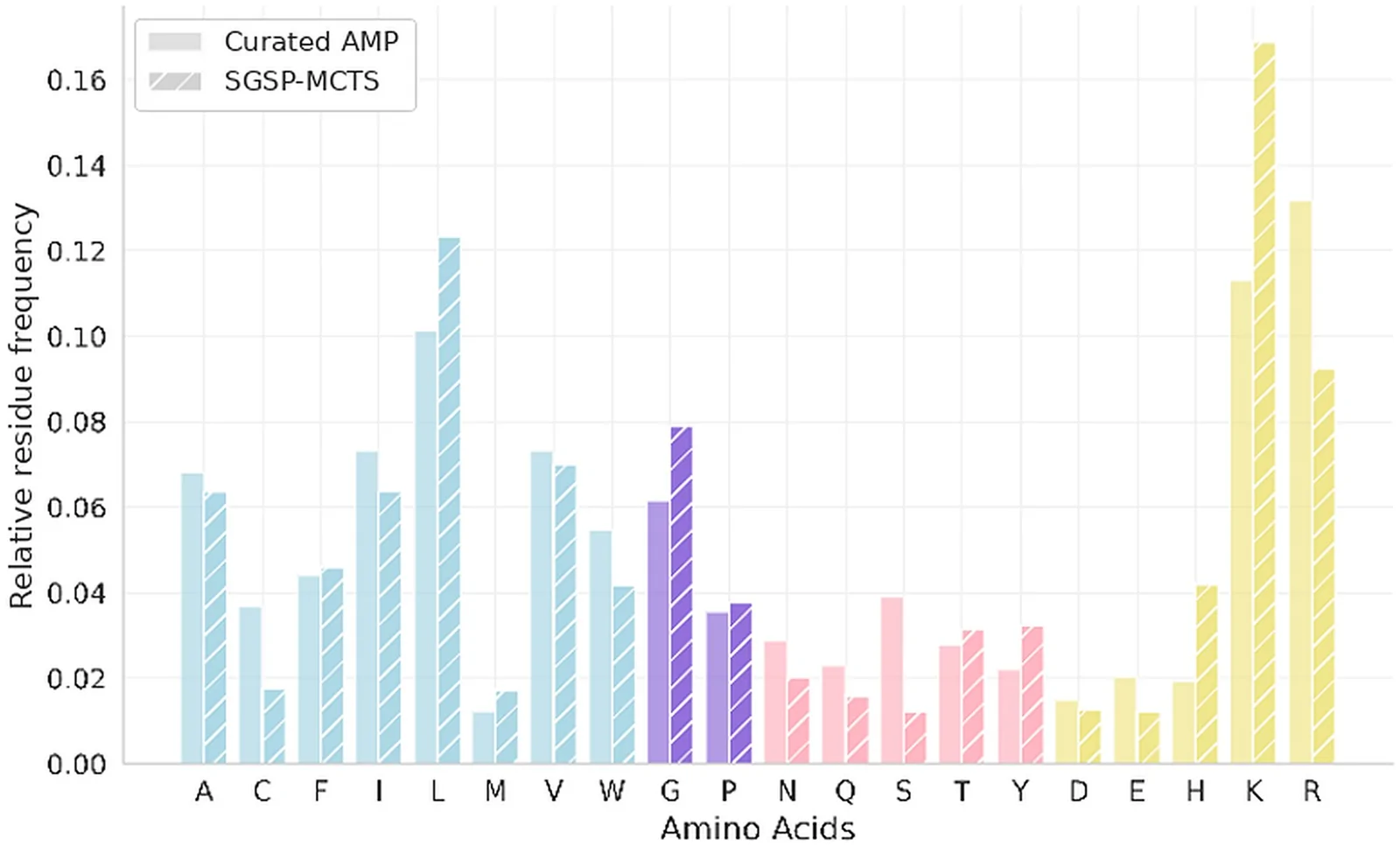
Amino-acid composition of curated AMP and SGSP-MCTS-generated sequence sets. Bars show the relative frequencies of the 20 standard amino acids in the curated AMP reference cohort and the SGSP-MCTS-generated peptide cohort. The analysis evaluates compositional correspondence and residue-frequency shifts between optimized sequences and established AMP sequences. AMP-versus-non-AMP classifier performance is evaluated separately in Supplementary Fig. S2 and Supplementary Table S2, while Fig. 5 provides complementary non-AMP physicochemical reference distributions.

### Candidate triage and structure-based *in silico* validation

Because candidate triage relies on AMP recognition beyond any single predictor, multiple AMP classifiers were benchmarked on an independent evaluation set. Supplementary Fig. S2 reports ROC curves and AUC values, and Supplementary Table S2 provides accuracy, precision, recall, and F1 summaries. These benchmarking results support the selection of a weighted consensus comprised of CAMP, PepNet, and AMPlify for downstream prioritization.

Shortlisted peptides were then evaluated by protein–peptide docking against five *S. aureus* targets. Figure 7 shows exemplar complexes and reports docking scores with associated confidence values, including MurA–AMP-1 with a docking score of −256.55 and confidence 0.8939, MurE–AMP-2 with a docking score of −272.89 and confidence 0.9211, FabG–AMP-3 with a docking score of −245.16 and confidence 0.8703, PBP2a–AMP-4 with a docking score of −275.62 and confidence 0.9250, and FtsZ–AMP-5 with a docking score of −245.12 and confidence 0.8702. The AMP-1 to AMP-5 labels in Fig. 7 refer to docking exemplars and are distinct from the synthesized peptides labeled AMP1 to AMP16 used in the *in vitro* assessment.

**Figure 7:**
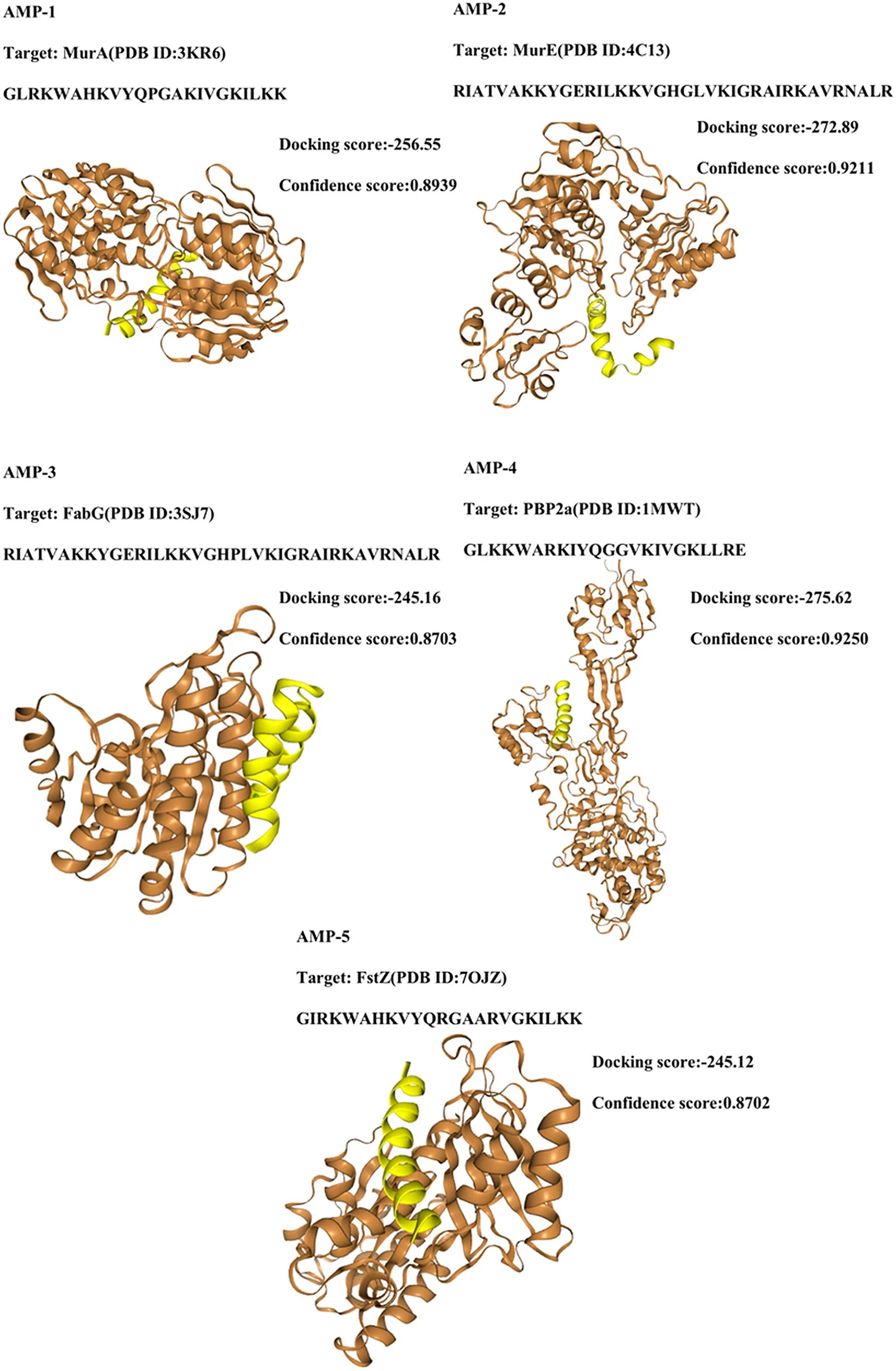
Representative docking models of peptide candidates against five *Staphylococcus aureus* protein targets. Representative peptide–protein complexes are shown for MurA, MurE, FabG, PBP2a, and FtsZ (PDB IDs indicated on the panels). Proteins are shown as cartoons and docked peptides are highlighted. Docking scores and confidence values correspond to the HDOCK outputs.

Membrane association and backbone conformational behavior were evaluated by MD simulations. Figure 8a reports membrane insertion using the S parameter, where the negative control PEX-F1-4 remains near zero over time while the positive control Hydraganan-1 and several tested candidates reach higher sustained values. Figure 8b reports backbone RMSD profiles, showing larger fluctuations for PEX-F1-4 and comparatively lower or more stable RMSD traces for several candidates.

**Figure 8:**
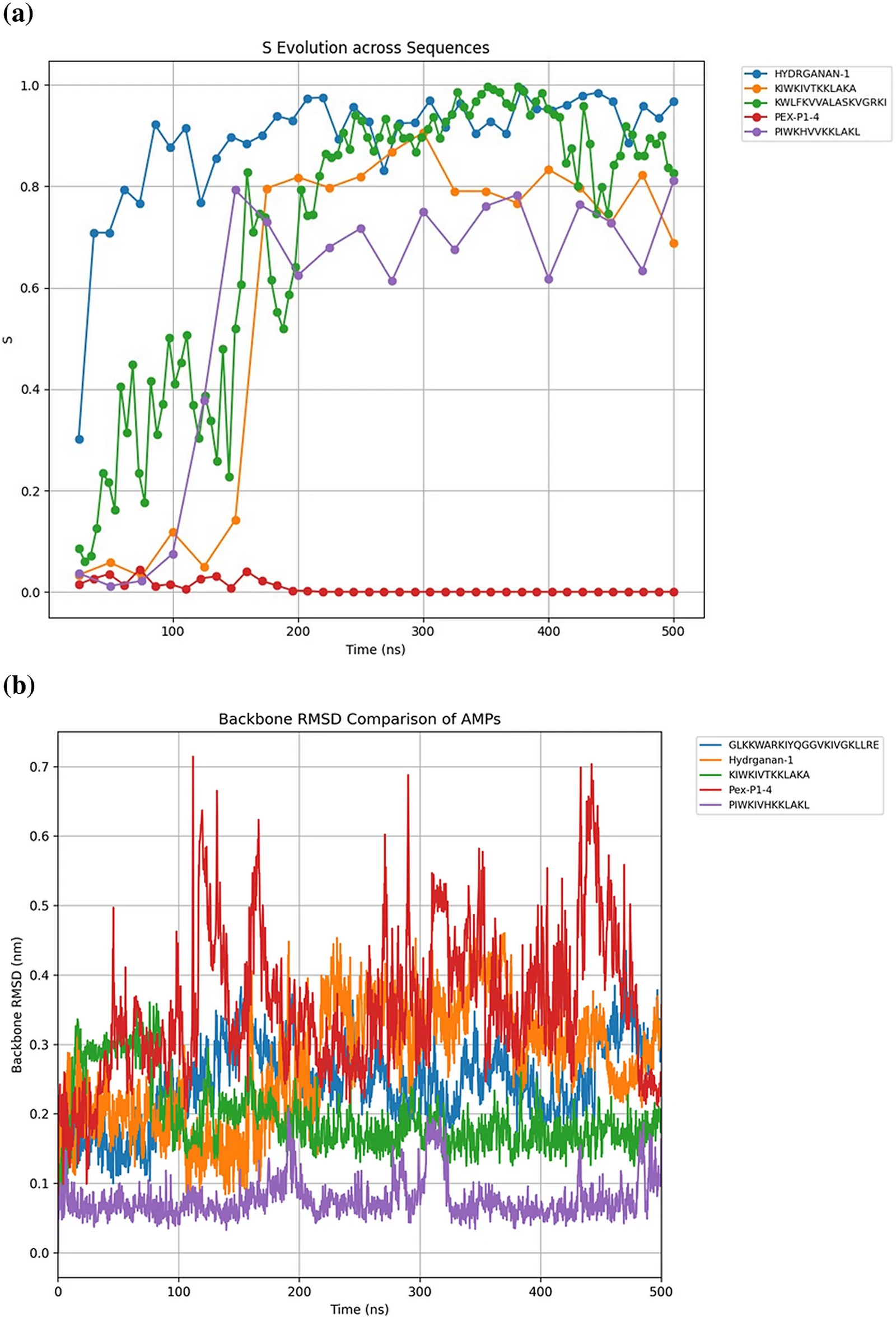
Membrane MD analyses of selected peptides. Panel (a) shows the time evolution of the membrane insertion parameter S during simulations in a POPE/POPG bilayer for selected candidates and controls. Panel (b) shows backbone RMSD trajectories relative to the starting conformation, describing simulated backbone conformational behavior over the 500-ns simulation window. This analysis does not represent an experimental peptide-stability assay.

### Benchmarking, novelty, and diversity analyses

Direct computational benchmarking was conducted for MCMC, PyAMPA, and SGSP-MCTS using the available optimization records. Figure 9a compares their optimization trajectories, while Table 1 reports the fractions of generated sequences satisfying the common benchmark criteria of predicted MIC <8 μg/ml, predicted hemolysis score/probability <0.5, and both criteria jointly. SGSP-MCTS achieved corresponding fractions of 0.949, 0.983, and 0.927; MCMC achieved 0.791, 0.398, and 0.328; and PyAMPA achieved 0.274, 0.904, and 0.250.

**Figure 9:**
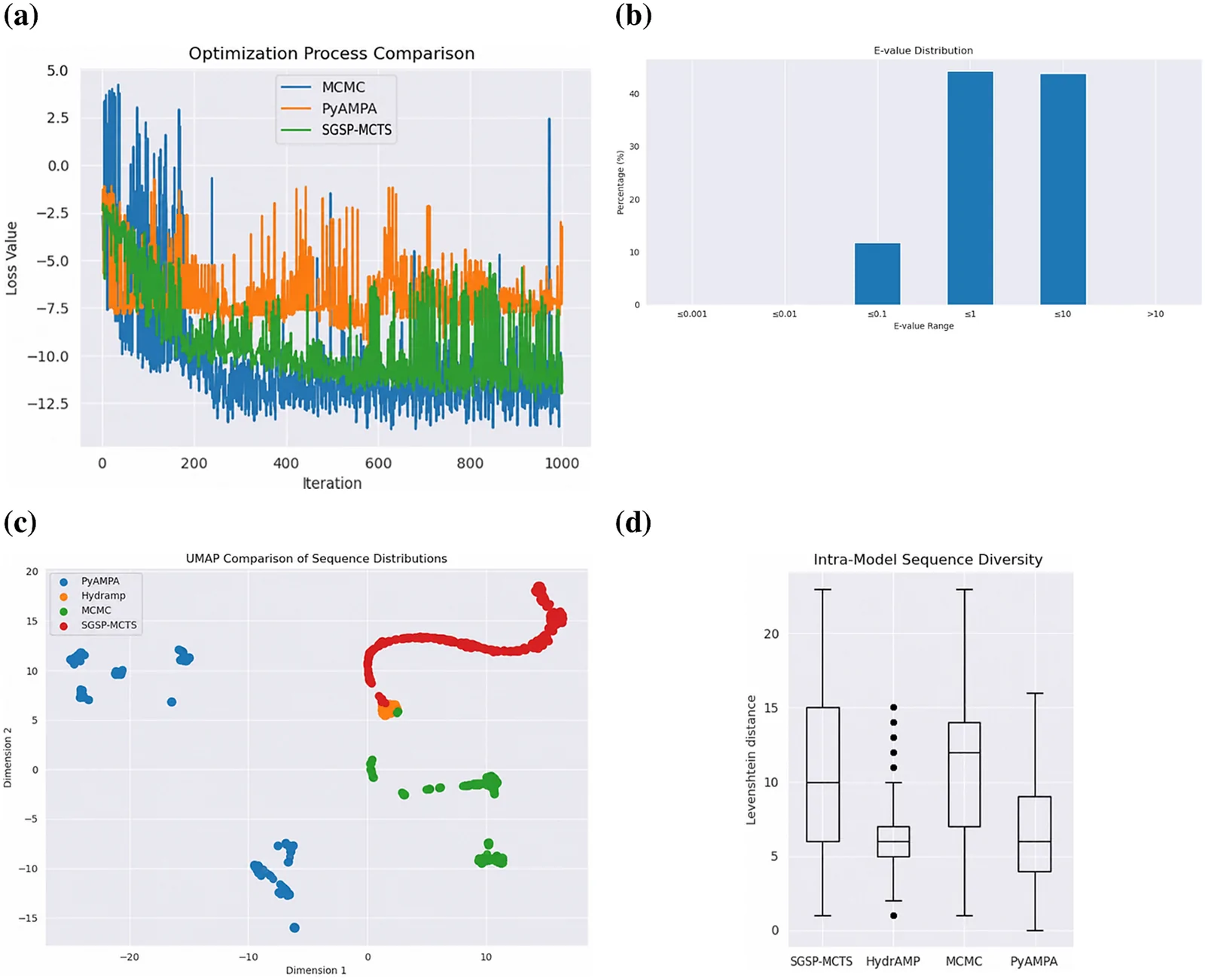
Cross-method computational analysis of optimization dynamics, novelty, and sequence diversity. Panel (a) compares optimization trajectories for MCMC, PyAMPA, and SGSP-MCTS initialized from the common sequence LFNRRGELRAIHKLKFWTLKDV. Panel (b) summarizes BLASTP E-value distributions for SGSP-MCTS-generated sequences aligned against dbAMP. Panel (c) shows ESM-2 embedding-based UMAP projections for available sequence sets from PyAMPA, HydrAMP, MCMC, and SGSP-MCTS. Panel (d) reports intra-method pairwise Levenshtein-distance distributions for the same four methods. The sequence sets in panels (c) and (d) were generated from the common starting sequence FLPMLAGLAANFLPKIVCKITKKC. The panels address complementary comparison levels and are interpreted separately.

**Table 1:** Surrogate-defined feasibility fractions for computationally generated sequences. Values represent the fractions of generated sequences satisfying predicted MIC <8 μg/ml, HemoDL-predicted hemolysis score/probability <0.5, and both criteria jointly.

Method	MIC (<8 μg/ml)	HEMO (<0.5)	Combination
SGSP-MCTS	0.949	0.983	0.927
MCMC	0.791	0.398	0.328
PyAMPA	0.274	0.904	0.250

Note: These fractions quantify computational enrichment under common predictor-based criteria. The hemolysis threshold of <0.5 was used for cross-method benchmarking, whereas the SGSP-MCTS candidate-triage workflow applied the more stringent threshold of <0.0001. Experimental agar-dilution outcomes are reported separately in Table 2. The SGSP-MCTS results were obtained using the fixed reward configuration reported in the Materials and Methods.

The predicted hemolysis threshold of <0.5 was used to provide a common cross-method feasibility benchmark. Candidate triage within the SGSP-MCTS workflow used the more stringent threshold of <0.0001 described in the Materials and Methods. The two thresholds therefore serve different analytical purposes.

Figure 9b summarizes BLASTP-based novelty of the SGSP-MCTS-generated sequences, whereas Fig. 9c and d describe embedding-space distributions and intra-method sequence diversity for the available HydrAMP, MCMC, PyAMPA, and SGSP-MCTS sequence sets. These analyses characterize computational behavior and sequence-space properties and are reported separately from the agar-dilution outcomes.

### *In vitro* agar dilution minimum inhibitory concentration testing of synthesized candidates

To connect computational sequence optimization and staged *in silico* validation with an experimental antibacterial endpoint, 16 peptides (AMP1–AMP16) were synthesized and evaluated by agar dilution MIC testing against a panel of 37 bacterial strains. Plate outcomes were interpreted using three qualitative categories—complete inhibition, partial inhibition, and no effect—based on colony formation patterns on Mueller–Hinton agar, as defined in Supplementary Table S3. Supplementary Table S4 provides the sequences of AMP1–AMP16 and summarizes the *in silico* prioritization metrics associated with their selection, including docking scores against five *S. aureus* targets, membrane MD stability, and hydrophobic/hydrophilic composition.

A representative strain-position map and example agar dilution plates for the three most active candidates are shown in Fig. 10, where panels b–d display AMP2, AMP7, and AMP9 at 32 and 64 μg/ml, respectively. Across the full concentration series tested up to 64 μg/ml, most peptides showed MIC values exceeding 64 μg/ml without detectable inhibition. A peptide-level MIC summary and the corresponding inhibited strains is reported in Table 2. The 16 synthesized peptides represented the final prioritized cohort selected from the larger SGSP-MCTS-generated population through staged computational triage. Complete inhibition of at least one tested strain within the 0.25–64 μg/ml range was used as the candidate-level experimental hit criterion. AMP2, AMP7, and AMP9 met this criterion, corresponding to 3 of 16 candidates (18.75%). AMP12 and AMP13 showed partial growth reduction against *Staphylococcus epidermidis* without complete inhibition within the tested range. The remaining 11 candidates showed no complete inhibition. The 18.75% fraction summarizes the observed yield of the prioritized experimental cohort and does not estimate the experimental activity of the complete generated sequence population. AMP2 showed strain-dependent inhibition between 16 and 64 μg/ml, with partial inhibition observed for *S. aureus* SA80, *S. aureus* SA1044, and *S. epidermidis*, and complete inhibition observed for *S. aureus* SA1209 and *E. coli* ATCC BAA-25922. AMP7 exhibited inhibition between 16 and 64 μg/ml, with partial inhibition of SA1044 and *S. epidermidis* and complete inhibition of *E. coli* ATCC BAA-25922. AMP9 exhibited MIC 64 μg/ml, with partial inhibition of *S. epidermidis* and complete inhibition of *E. coli* ATCC BAA-25922. Two additional peptides, AMP12 and AMP13, were recorded as MIC >64 μg/ml with partial inhibition noted for *S. epidermidis*, while the remaining peptides showed MIC >64 μg/ml without detectable inhibition under the assay conditions.

**Figure 10:**
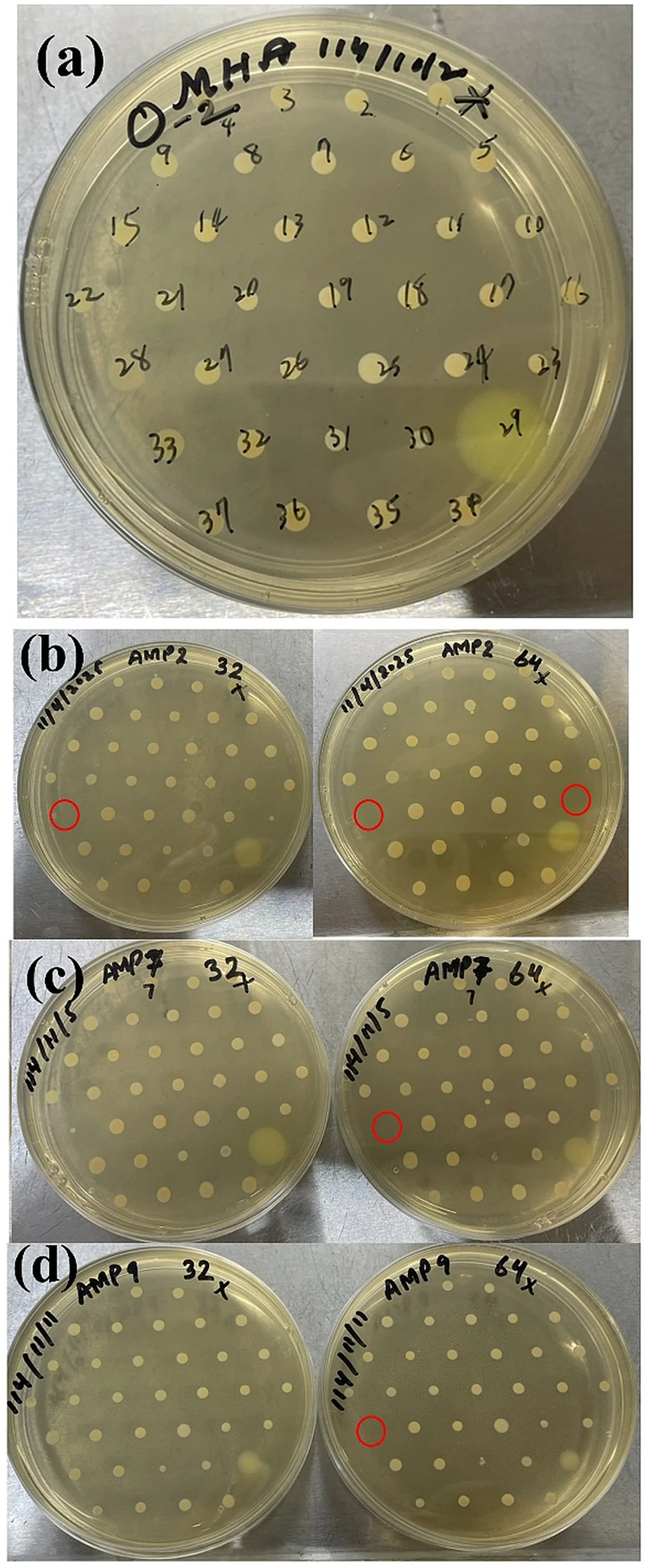
Agar dilution antibacterial assessment of AMP2, AMP7, and AMP9 across a 37-strain panel. Panel (a) shows the strain–position map on Mueller–Hinton agar without peptide, where each numbered inoculation spot corresponds to a strain ID used throughout the study. Panels (b–d) show representative agar dilution plates containing AMP2, AMP7, or AMP9 at 32 and 64 μg/ml, inoculated using a multi-pin replicator and incubated overnight at 37°C. Circles indicate strains showing complete inhibition at the indicated concentration, defined as no visible colony growth relative to the peptide-free control. The 37-strain panel comprised 25 *Staphylococcus aureus* isolates from this study (IDs 1–25), five reference strains (IDs 26–30), and seven clinical *Staphylococcus* isolates (IDs 31–37) including *Staphylococcus epidermidis*.

**Table 2:** Agar-dilution outcomes for the 16 SGSP-MCTS-prioritized peptides. Strain-specific MICs and partial growth-reduction observations were recorded across the 37-strain panel over the 0.25–64 μg/ml concentration range.

Peptide	Observed strain-specific MIC or tested-range outcome (μg/mL)	Strains showing inhibition within tested range
AMP2	16–64	**Partial inhibition:** *S. aureus* SA80; *S. aureus* SA1044; *S. epidermidis* **Complete inhibition:** *S. aureus* SA1209; *E. coli* ATCC BAA-25922
AMP7	16–64	**Partial inhibition:** *S. aureus* SA1044; *S. epidermidis* **Complete inhibition:** *E. coli* ATCC BAA-25922
AMP9	64	**Partial inhibition:** *S. epidermidis* **Complete inhibition:** *E. coli* ATCC BAA-25922
AMP12	>64	**Partial inhibition:** *S. epidermidis*
AMP13	>64	**Partial inhibition:** *S. epidermidis*
AMP1	>64	No inhibited strains observed
AMP3	>64	No inhibited strains observed
AMP4	>64	No inhibited strains observed
AMP5	>64	No inhibited strains observed
AMP6	>64	No inhibited strains observed
AMP8	>64	No inhibited strains observed
AMP10	>64	No inhibited strains observed
AMP11	>64	No inhibited strains observed
AMP14	>64	No inhibited strains observed
AMP15	>64	No inhibited strains observed
AMP16	>64	No inhibited strains observed

Note: Complete inhibition was defined as no visible colony growth and was used to assign strain-specific MIC and candidate-level experimental hit status. AMP2, AMP7, and AMP9 achieved complete inhibition of at least one tested strain, corresponding to 3 of 16 candidates (18.75%). Partial growth reduction for AMP2, AMP7, AMP9, AMP12, and AMP13 was recorded separately.

## Discussion and conclusion

This study integrates SGSP-MCTS with staged *in silico* triage and agar dilution MIC testing to address two practical limitations in AMP discovery: efficient exploration of a large combinatorial sequence space under competing constraints, and the weak correspondence that often exists between computational prioritization and standardized *in vitro* antibacterial readouts. The core methodological contribution is the adaptation of an AlphaZero-style policy–value MCTS loop to peptide sequence optimization, enabling look-ahead planning over multi-step mutation trajectories rather than selecting edits by local, single-step improvements alone. Because SGSP-MCTS applies sequential single-site substitutions, each search trajectory is conditioned on the selected seed and its local sequence neighborhood. The starting pool was enriched for activity-related annotations and predictions by design, while the role of SGSP-MCTS was to explore multi-step variants and refine the joint computational profile of these promising scaffolds before post-generation eligibility and downstream prioritization.

The principal architectural distinction of SGSP-MCTS is its explicit policy–value-guided look-ahead planning over sequential amino-acid substitutions. At each sequence state, the policy head provides mutation priors, the value head estimates the expected sequence value, and MCTS repeatedly simulates subsequent sequence states before constructing the search-improved mutation distribution. This design provides a search mechanism that differs from one-pass generation and latent-space sampling used by GAN-, VAE-, and diffusion-based frameworks. The cross-method evidence in this study is interpreted according to the available comparison level. Direct optimization trajectories and surrogate-defined feasibility were evaluated for MCMC, PyAMPA, and SGSP-MCTS. Physicochemical properties were compared using available outputs from HydrAMP, Diff-AMP, latent diffusion, MCMC, PyAMPA, and SGSP-MCTS. Sequence-space and intra-method diversity were evaluated for HydrAMP, MCMC, PyAMPA, and SGSP-MCTS. AMPGAN v2, Multi-CGAN, and HMAMP were included for methodological positioning because matched output sets were unavailable in the retained study records. The scope of these comparisons is summarized in Supplementary Table S5. These analyses characterize the evaluated implementations and sequence sets and do not establish universal superiority across all AMP generation frameworks.

The components of the framework operate at distinct stages. MCTS and the policy–value network constitute the sequence-optimization engine. Within the fixed reward, ESMFold pLDDT and HemoDL provide auxiliary continuous guidance during tree search. Strict pLDDT and HemoDL thresholds subsequently determine post-generation eligibility before classifier-consensus and structure-based prioritization. The weighted CAMP, PepNet, and AMPlify consensus provides an independent AMP-recognition score for post-search ranking; its model selection is supported by the independent DBAASP benchmark in Supplementary Fig. S2 and Supplementary Table S2. Docking and membrane MD provide additional structural and membrane-interaction information for final candidate prioritization. All reported optimization, filtering, and candidate-selection outcomes were obtained using this complete configuration. The current evidence therefore characterizes the integrated workflow and does not quantify the isolated contribution of an individual component.

The agar-dilution experiment provided an assay-specific assessment of the 16 candidates prioritized by the integrated computational workflow. Complete inhibition of at least one tested strain within the 0.25–64 μg/ml concentration range was observed for AMP2, AMP7, and AMP9, corresponding to 3 of 16 synthesized candidates (18.75%). AMP12 and AMP13 showed partial growth reduction against *S. epidermidis* without complete inhibition within the tested range, whereas the remaining 11 candidates showed no complete inhibition. These findings show that the staged workflow identified a small experimentally active subset and also reveal a substantial gap between surrogate-defined feasibility and measured antibacterial activity. The surrogate pass fractions and the 18.75% candidate-level complete-inhibition yield represent different sequence populations and different endpoints and are therefore interpreted separately. The difference between computational screening and agar-dilution activity indicates that the current surrogate objectives do not capture all determinants of assay-specific antibacterial performance. Assay conditions, peptide handling, and strain-dependent biological factors may contribute to this discrepancy; these factors were not directly evaluated in the present study and are therefore considered directions for subsequent investigation.

The present study did not include matched HydrAMP, PyAMPA, MCMC, or random-selection cohorts that were selected, synthesized, and evaluated using the same candidate-selection procedure, 37-strain panel, Mueller–Hinton agar-dilution protocol, concentration range, and candidate-level complete-inhibition criterion. Experimental hit fractions are influenced by the number and selection of synthesized peptides, bacterial species and strains, assay format, concentration range, and activity definition. Values obtained from separate studies would therefore reflect differences in experimental design together with differences between the computational methods. We did not construct a cross-study quantitative ranking of experimental hit rates. The current cross-method assessment is restricted to the physicochemical distributions in Fig. 5, surrogate-defined feasibility fractions in Table 1, and novelty and diversity analyses in Fig. 9. A prospective matched benchmark will be required to determine comparative experimental hit rates.

Some limitations follow directly from these results and motivate targeted next steps. First, the limited complete-inhibition yield among the prioritized candidates indicates the need for assay-aware recalibration of the activity surrogates and for uncertainty-aware candidate selection using experimentally measured outcomes. Future model refinement should more directly incorporate standardized assay data so that surrogate improvement corresponds more closely to antibacterial activity under the intended experimental conditions. Second, the experimental assessment in the present study was limited to agar-dilution antibacterial activity. This endpoint establishes bacterial growth inhibition under the tested conditions and does not determine selectivity over mammalian cells. HemoDL provided a computational estimate of hemolytic propensity; experimental hemolysis and broader mammalian-cell cytotoxicity were not measured. Membrane MD characterized simulated membrane association and backbone conformational behavior and does not establish experimental peptide stability. Direct hemolysis, mammalian-cell cytotoxicity, and assay-relevant peptide-stability testing remain necessary for subsequent validation. Third, the present study evaluates one fixed reward formulation within the complete integrated pipeline. The retained study records do not include systematic coefficient-perturbation, reward-term removal, or component-disabled runs for MCTS, policy–value guidance, classifier consensus, docking, or membrane MD. The current results therefore characterize the complete configuration and do not quantify the marginal or interactive contribution of individual components. Prospectively designed coefficient-sensitivity and component-wise ablation analyses will be required for this purpose. Finally, MCTS remains computationally more demanding than purely feed-forward generation; efficiency improvements through parallelization and policy/value distillation are important for scaling.

In conclusion, we developed an SGSP-MCTS framework that combines policy–value-guided tree search with multi-property surrogate evaluation and staged candidate prioritization. Under the evaluated computational criteria, the framework improved surrogate-defined multi-property scores and produced AMP-relevant physicochemical and sequence-space characteristics. Agar-dilution testing of 16 prioritized candidates identified AMP2, AMP7, and AMP9 as achieving complete inhibition of at least one tested strain, while AMP12 and AMP13 showed partial growth reduction without complete inhibition within the tested range. These results establish the current computational-to-experimental performance of the integrated workflow and quantify the remaining gap between surrogate-defined enrichment and assay-specific antibacterial activity. The present evidence supports SGSP-MCTS as a computational sequence-optimization and candidate-prioritization framework under the evaluated settings. Comparative experimental advantage over alternative approaches will require a prospectively matched study in which candidates are selected, synthesized, and evaluated under identical experimental conditions.

**Key Points**

- A surrogate-guided self-play Monte Carlo tree search framework is introduced to optimize antimicrobial peptide sequences under concurrent predicted-activity, HemoDL-predicted hemolysis, and structural-confidence criteria.
- The approach integrates policy–value learning with property surrogates and classifier consensus, followed by docking and membrane molecular dynamics to prioritize candidates for synthesis.
- Cross-method evaluation combines direct optimization benchmarking against MCMC and PyAMPA with broader physicochemical and sequence-diversity comparisons using available outputs from HydrAMP, Diff-AMP, latent diffusion, MCMC, and PyAMPA.
- Agar-dilution testing of 16 prioritized candidates identified three peptides achieving complete inhibition of at least one tested strain and two additional peptides showing partial growth reduction.

## Supplementary Material

Supplementary_Materials_bbag618

## Data Availability

The sequences of the 16 synthesized peptides and the reported antibacterial assay outcomes are provided in Supplementary Table S4 and Table 2, respectively. The public peptide resources used in this study are described in the Materials and Methods section [19, 25, 26]. The SGSP-MCTS reference implementation for model training and peptide sequence search, together with selected supporting data and execution instructions, is available at https://github.com/ncubiomedlab/SGSP-MCTS/. The repository documentation describes the contents and limitations of the public release.
