# Supplementary material for: Surrogate-guided self-play Monte Carlo tree search for antimicrobial peptide sequence optimization and in vitro antibacterial assessment
Source: Brief Bioinform. 2026 Nov 13;27(6):bbag618. doi: 10.1093/bib/bbag618 (PMC13759990; doi:10.1093/bib/bbag618)
Supplement: Supplementary file 1 [file supplementary_materials_bbag618.pdf]

Surrogate-Guided Self-Play Monte Carlo Tree Search for Antimicrobial Peptide Sequence Optimization and *In Vitro* Antibacterial Assessment

Jang-Jih Lu[1,†], Chia-Ru Chung[2,†], Chen-Hao Chang[2], Li-Ching Wu[2,3], Justin Bo-Kai Hsu[4], Cheng-Yu Tsai[1], Tzong-Yi Lee[5,6,*] and Jorng-Tzong Horng[2,*]

[1]Division of Clinical Pathology, Taipei Tzu Chi Hospital, Buddhist Tzu Chi Medical Foundation, New Taipei City, Taiwan

[2]Department of Computer Science and Information Engineering, National Central University, Taoyuan, Taiwan

[3]Department of Biomedical Sciences and Engineering, National Central University, Taoyuan, Taiwan

[4]Department of Computer Science and Engineering, Yuan Ze University, Taoyuan, Republic of China

[5]Institute of Bioinformatics and Systems Biology, National Yang Ming Chiao Tung University, Hsinchu, Taiwan

[6]Center for Intelligent Drug Systems and Smart Biodevices (IDS2B), National Yang Ming Chiao Tung University, Hsinchu City, Taiwan

†These authors contributed equally to this work.

*Corresponding author. Department of Computer Science and Information Engineering, National Central University, No. 300, Zhongda Rd., Zhongli District, Taoyuan City, Taiwan. E-mail: horng@db.csie.ncu.edu.tw

Supplementary Methods

Peptide handling and dilution calculations for agar dilution MIC

For agar-dilution plate preparation, each peptide was dissolved in sterile water at 8 mg/mL as a concentrated assay stock (e.g., 9 mg in 1.125 mL). The stock was divided into 0.3-mL aliquots in low-binding tubes and stored at −80°C. Working aliquots were maintained at 4°C during plate preparation.

Final agar concentrations were 0.25, 0.5, 1, 2, 4, 8, 16, 32, and 64 µg/mL. For the 16, 32, and 64 µg/mL plates, 50, 100, and 200 µL of the 8 mg/mL stock, respectively, were added to 25 mL molten agar. Intermediate peptide solutions were used for the lower concentrations.

Following gentle mixing, agar was poured into sterile plates, allowed to solidify, and dried before inoculation. Long-term stability of peptide stocks and peptide-containing agar was not experimentally assessed.

Bacterial strain panel and inoculum preparation

The assay used 37 strains:

- 25 prior *S. aureus* isolates (IDs 1–25): SA2, SA6, SA9 (yellow), SA10, SA22, SA33, SA80, SA82, SA241, SA1019, SA236, SA250, SA1021, SA1030, SA1033, SA1040, SA1043, SA1044, SA1045, SA1049, SA1054, SA1063, SA1209, SA1215, SA9 (white).
- Five reference strains (IDs 26–30): *S. aureus* ATCC 1026; *S. aureus* ATCC (as provided); *E. coli* ATCC BAA-25922; *P. aeruginosa* ATCC 27853; *Enterococcus* reference strain (as provided).
- Seven clinical *Staphylococcus* isolates (IDs 31–37): *Staphylococcus epidermidis*, clinical S.A_1_S, S.A_2_S, S.A_3_R, S.A_5_S, S.A_6_R, S.A_7_R, where S/R indicate vancomycin susceptible/resistant.

All strains were cultured overnight at 37°C on blood agar plates (BAP). A single colony was suspended in saline and adjusted to 0.5 McFarland using spectrophotometry prior to agar dilution inoculation.

Supplementary Figures

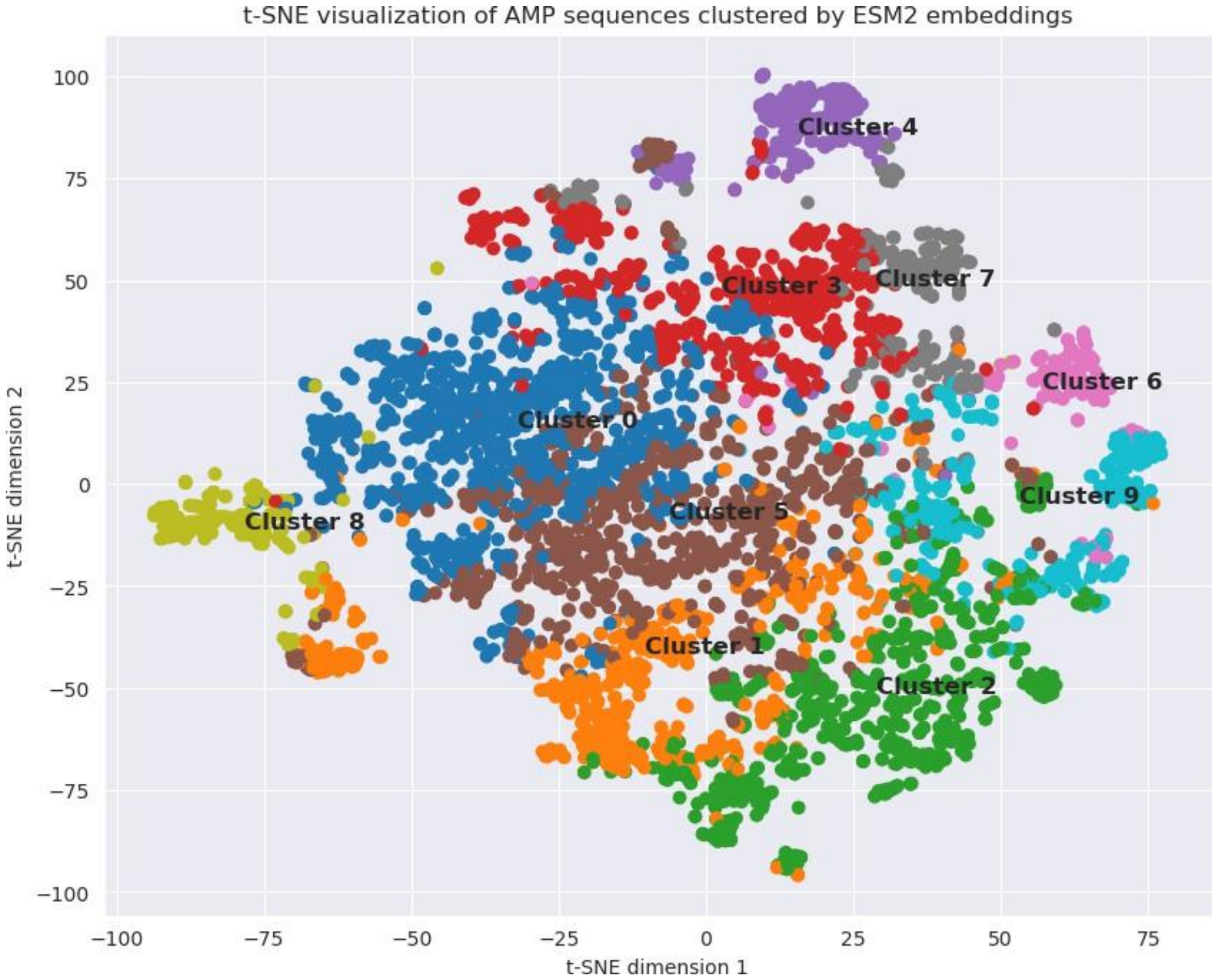

Supplementary Figure S1. Diversity-controlled selection of dbAMP representatives using ESM-2 embeddings. dbAMP sequences were embedded using ESM-2 representations and partitioned by K-means clustering with k=10. Five representative sequences were selected from each cluster, yielding 50 diversity-controlled dbAMP sequences that were subsequently combined with HydrAMP measured-MIC peptides to form the optimization starting pool.

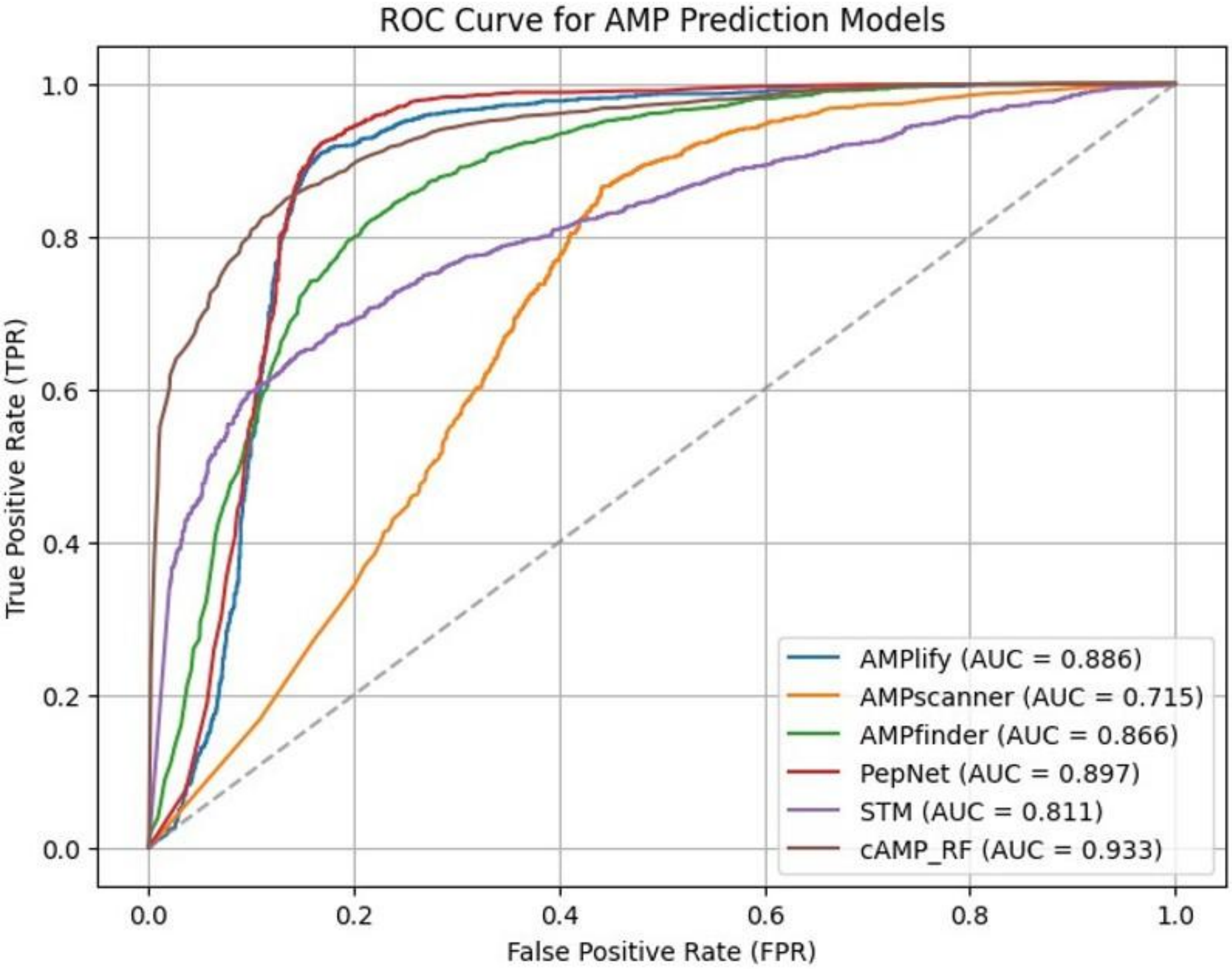

Supplementary Figure S2. ROC curves for AMP prediction models used for classifier consensus. Receiver operating characteristic curves and AUC values compare CAMP, AMPlify, PepNet, STM, AMPscanner, and AMPfinder on the independent test set. CAMP, PepNet, and AMPlify were selected for weighted integration. Additional summary metrics are reported in **Supplementary Table S1**.

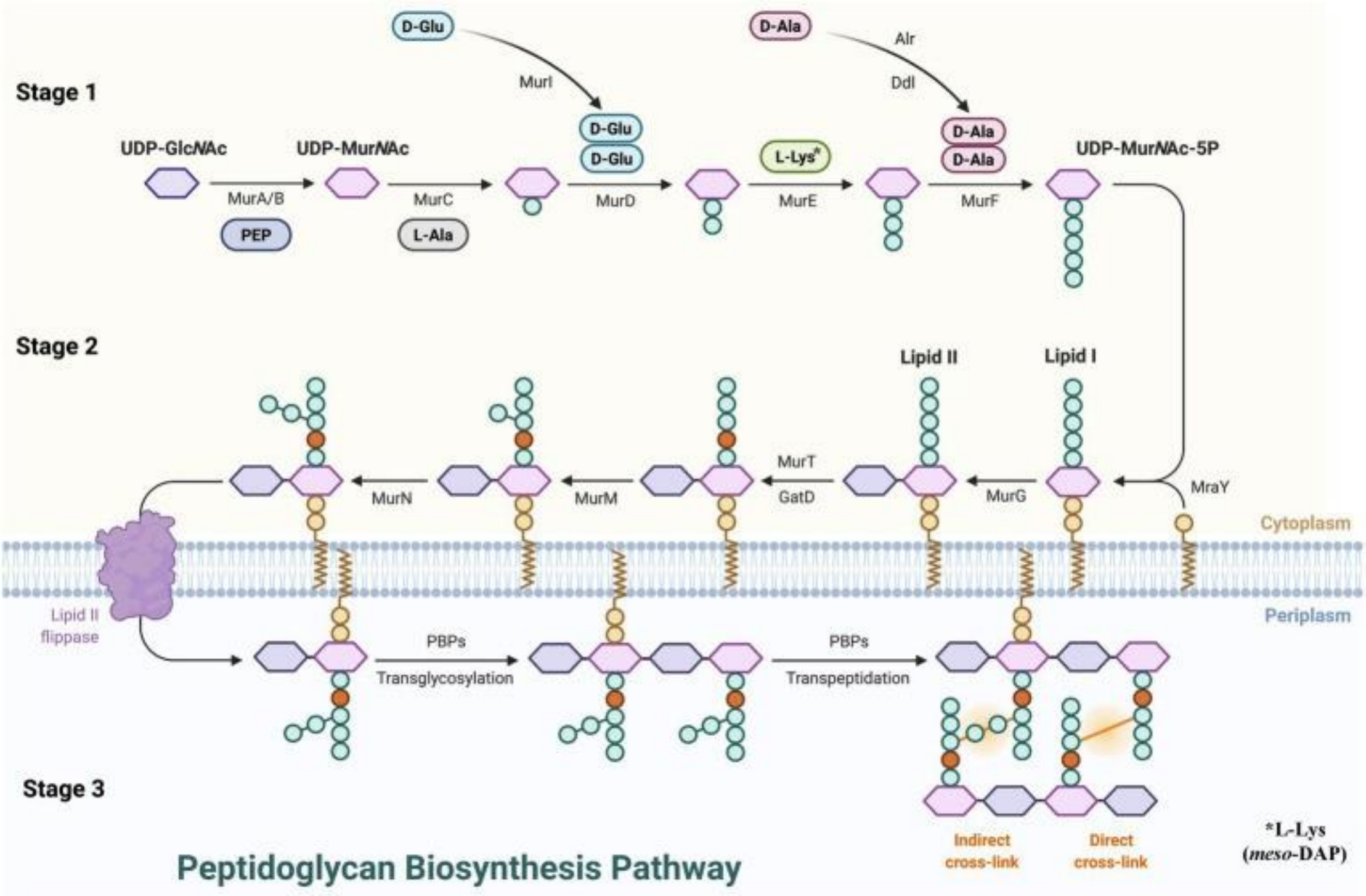

Supplementary Figure S3. Peptidoglycan biosynthesis pathway. The schematic shows cytoplasmic precursor synthesis, membrane-associated lipid intermediates, and peptidoglycan polymerization and cross-linking. MurA/MurB, MurE, and penicillin-binding proteins (PBPs) are indicated. Reproduced from Figure 1 of Zhou et al. [36] (© 2022 the authors), under the Creative Commons Attribution 4.0 International License (https://creativecommons.org/licenses/by/4.0/).

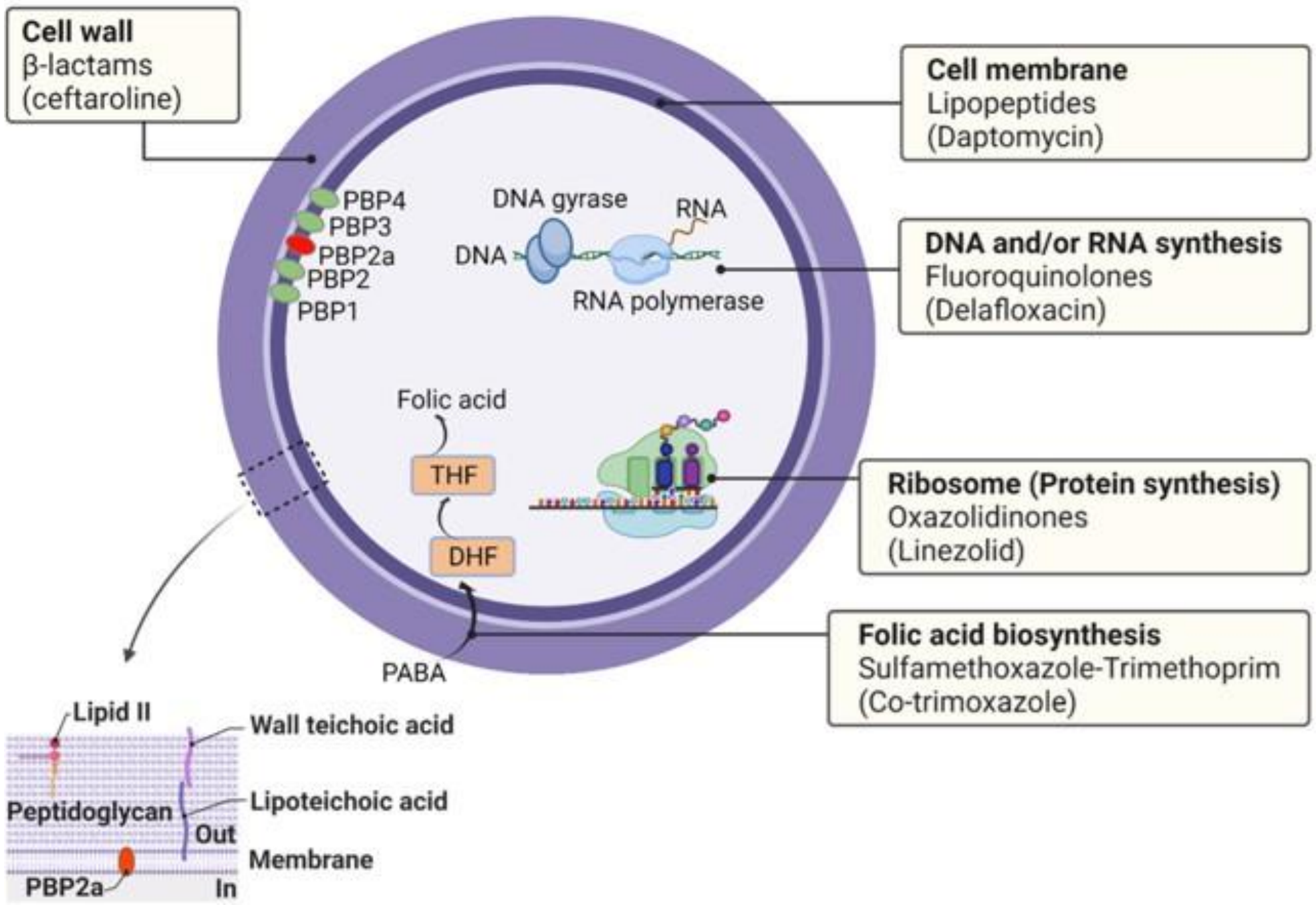

Supplementary Figure S4. Major antibiotic target classes in Staphylococcus aureus. The schematic summarizes antibacterial targets involving the cell wall, cell membrane, nucleic acid synthesis, ribosomes, and folic acid biosynthesis, providing context for receptor selection. Reproduced from Figure 2 of Lade and Kim [35] (© 2021 the authors), under the Creative Commons Attribution 4.0 International License (https://creativecommons.org/licenses/by/4.0/).

Supplementary Table S1. Fixed components and coefficients of the SGSP-MCTS scalar reward.

Component	Expression in reward	Fixed coefficient	Numerical scale	Intended role
AMPActiPred MIC-oriented output	(1/(1+MIC))	5.0	Greater than 0 and no greater than 1 when MIC is defined	Prioritize lower predicted MIC values
ESMFold pLDDT	pLDDT	0.02	Approximately 0–100	Provide auxiliary structural-confidence guidance during search; final eligibility requires pLDDT > 60.
MIC-support classifier	A	1.0	Probability-like output, 0–1	Maintain an activity-oriented signal when AMPActiPred does not return an MIC estimate
HemoDL hemolysis score	-H	2.0	Probability-like output, 0–1	Provide an auxiliary penalty for predicted hemolytic propensity during search; final eligibility requires HemoDL score < 0.0001.
N-gram KL divergence		0.1	Non-negative and data-dependent	Limit excessive departure from high-activity AMP motif statistics

Note: All reported SGSP-MCTS optimization analyses used the complete fixed reward configuration shown above. The table summarizes the numerical formulation and intended role of each reward component. It does not represent a coefficient-sensitivity or component-ablation analysis. HemoDL values are computational predictions of hemolytic propensity and do not constitute experimental hemolysis or broader mammalian-cell cytotoxicity measurements.

Supplementary Table S2. Performance metrics of AMP prediction models on the independent test set. Accuracy, precision, recall, and F1-score are reported for AMPlify, AMPscanner, AMPfinder, PepNet, STM, and CAMP on the independent evaluation set used to select models for weighted classifier consensus.

Model	Accuracy	Precision	Recall	F1-score
AMPlify	0.858	0.857	0.860	0.858
AMPscanner	0.704	0.659	0.845	0.741
AMPfinder	0.756	0.686	0.946	0.795
PepNet	0.872	0.852	0.901	0.876
STM	0.745	0.776	0.689	0.730
CAMP	0.841	0.796	0.917	0.852

Supplementary Table S3. Plate-growth scoring criteria for agar dilution MIC testing. Definitions used to score bacterial growth on Mueller–Hinton agar plates, including complete inhibition, partial inhibition, and no effect, based on colony formation relative to the peptide-free control.

**Growth category**	**Observed growth on MHA plate**
Complete inhibition	No visible colonies
Partial inhibition	Few colonies or sparse colony growth
No effect	Colony growth comparable to the control plate

Note: Only complete inhibition was used to determine the strain-specific MIC and candidate-level experimental hit status. Partial inhibition indicates reduced colony growth and was reported as a separate qualitative observation.

Supplementary Table S4. Final SGSP-MCTS-prioritized peptide cohort selected for experimental testing. The table lists the 16 synthesized peptides and their available docking, membrane MD, and physicochemical prioritization descriptors. These peptides constitute the final experimental cohort selected from the larger SGSP-MCTS-generated sequence population through staged computational triage. Sequences of the 16 synthesized peptides (AMP1–AMP16) and their associated in silico prioritization metrics, including HDOCK docking scores against five *S. aureus* targets (PBP2a, MurA, FabG, MurE, and FtsZ), the docking score, membrane MD backbone RMSD, and hydrophobic/hydrophilic residue counts and percentages.

	Sequence	PBP2a	MurA	FabG	MurE	SaFtsZ	Docking	RMSD	Hydrophobic	Hydrophilic	Hydrophobic%	Hydrophilic%
AMP1	GLKKHARKVYQGGVKIVGKLLRA	-264.44	-217.16	-207.58	-235	-210.37	-1134.6	0.4819	13	10	56.50%	43.50%
AMP2	GLKKWARKIYQGGVKIVGKLLRE	-275.62	-202.2	-208.81	-217.7	-201.5	-1105.8	0.2443	13	10	56.50%	43.50%
AMP3	GIRKWAHKVYQRGAARVGKILKK	-237.23	-216	-227.2	-250.1	-245.12	-1175.7	0.4289	12	11	52.20%	47.80%
AMP4	GLRKWAHKVYQRGVKPVGKILKK	-240.12	-217.04	-233.59	-215.88	-207.48	-1114.1	0.4776	12	11	52.20%	47.80%
AMP5	GIKKWVKHVYQGGVKIVGKLLRA	-239.39	-213.45	-219.48	-247.19	-228.61	-1148.1	0.3963	14	9	60.90%	39.10%
AMP6	GLKKWAHKVYQGGVKIVGKILPK	-235.23	-198.22	-203.4	-247.4	-199.91	-1084.2	0.4376	14	9	60.90%	39.10%
AMP7	GLKKWVRKITQGGVKIVGKLLRA	-231.2	-220.59	-216.62	-220.23	-200.69	-1089.3	0.1573	14	9	60.90%	39.10%
AMP8	PIWKIVHKKLAKL	-221.67	-169.97	-178.43	-238.66	-181.34	-990.07	0.077	8	5	61.50%	38.50%
AMP9	KIWKSVVKKLLKA	-217.46	-167	-178.57	-217.87	-185.46	-966.36	0.2357	7	6	53.80%	46.20%
AMP10	PIWKHVVKKLAKL	-209.29	-165.29	-178.38	-205.05	-188.89	-946.9	0.2233	8	5	61.50%	38.50%
AMP11	KIWKSVVKKLIKA	-211.64	-189.41	-178.16	-186.62	-175.87	-941.7	0.311	7	6	53.80%	46.20%
AMP12	PIWKIRVKKLAKL	-203.09	-199.98	-190.79	-194.27	-172.39	-960.52	0.3476	8	5	61.50%	38.50%
AMP13	RIWKIPVKKIAKL	-197.64	-178.85	-185.08	-212.88	-170.35	-944.8	0.3873	8	5	61.50%	38.50%
AMP14	RIWKIVPKKLAKL	-195.96	-174.64	-196.91	-196.98	-184.58	-949.07	0.208	8	5	61.50%	38.50%
AMP15	KIWKIVTKKLAKA	-184.97	-173.72	-173.3	-184.18	-171.14	-887.31	0.1958	7	6	53.80%	46.20%
AMP16	PIWKIKVKKLAKL	-195.69	-163.7	-177.04	-179.97	-166	-882.4	0.2296	8	5	61.50%	38.50%

Supplementary Table S5. Scope of cross-method comparisons included in the present study.

Method	Framework or principal role	Direct optimization trajectory and feasibility comparison	Physicochemical comparison	UMAP and intra-method diversity comparison	Evidence level in the present study
AMPGAN v2	Conditional GAN-based AMP generation	No	No	No	Methodological positioning
Multi-CGAN	Multi-property conditional GAN	No	No	No	Methodological positioning
HMAMP	Hypervolume-driven multi-objective generative framework	No	No	No	Methodological positioning
HydrAMP	Conditional VAE-based AMP generation and optimization	No	Figure 5	Figure 9 (c)–(d)	Sequence-level physicochemical and diversity comparison
Diff-AMP	Integrated AMP generation, prediction, and optimization framework	No	Figure 5	No	Sequence-level physicochemical comparison
Latent diffusion	Latent-space diffusion-based AMP generation	No	Figure 5	No	Sequence-level physicochemical comparison
MCMC	Stochastic sequence-optimization baseline	Figure 9 (a) and Table 1	Figure 5	Figure 9 (c)–(d)	Direct optimization and sequence-level comparison
PyAMPA	Prediction and genetic-algorithm-based optimization tool	Figure 9 (a) and Table 1	Figure 5	Figure 9 (c)–(d)	Direct optimization and sequence-level comparison
SGSP-MCTS	Policy–value-guided MCTS sequence optimization	Figure 9 (a) and Table 1	Figure 5	Figure 9 (c)–(d)	Proposed integrated framework
